# Venom alkaloids against Chagas disease parasite: search for effective therapies

**DOI:** 10.1038/s41598-020-67324-8

**Published:** 2020-06-30

**Authors:** Rafael C. M. Costa Silva, Eduardo G. P. Fox, Fabio M. Gomes, Daniel F. Feijó, Isabela Ramos, Carolina M. Koeller, Tatiana F. R. Costa, Nathalia S. Rodrigues, Ana P. Lima, Georgia C. Atella, Kildare Miranda, Alejandra C. Schoijet, Guillermo D. Alonso, Ednildo de Alcântara Machado, Norton Heise

**Affiliations:** 1 Instituto de Biofísica Carlos Chagas Filho, Centro de Ciências da Saúde, Universidade Federal do Rio de Janeiro, Rio de Janeiro, RJ 21941-902 Brazil; 2 Instituto de Microbiologia Paulo de Góes, Centro de Ciências da Saúde, Universidade Federal do Rio de Janeiro, Rio de Janeiro, RJ 21941-902 Brazil; 3 Laboratory of Malaria and Vector Research, National Institute of Allergy and Infectious Diseases, National Institutes of Health, Rockville, MD 20852 USA; 4 Instituto de Bioquímica Médica Leopoldo de Meis, Centro de Ciências da Saúde, Universidade Federal Do Rio de Janeiro, Rio de Janeiro, RJ 21941-902 Brazil; 5 Instituto Nacional de Ciência e Tecnologia em Entomologia Molecular, Rio de Janeiro, RJ 21941-902 Brazil; 6 Department of Microbiology and Immunology, School of Medicine and Biological Sciences, University at Buffalo, Buffalo, NY 14203 USA; 7 Instituto Nacional de Ciência e Tecnologia em Biologia Estrutural e Bioimagem, Rio de Janeiro, RJ 21941-902 Brazil; 8 Instituto de Investigaciones en Ingeniería Genética y Biología Molecular “Dr. Héctor N. Torres” (INGEBI-CONICET), C1428ADN Buenos Aires, Argentina; 9 Red Imported Fire Ant Research Centre, South China Agricultural University, Guangzhou, 510642 People’s Republic of China

**Keywords:** Antimicrobials, Parasitology

## Abstract

Chagas disease is an important disease affecting millions of patients in the New World and is caused by a protozoan transmitted by haematophagous kissing bugs. It can be treated with drugs during the early acute phase; however, effective therapy against the chronic form of Chagas disease has yet to be discovered and developed. We herein tested the activity of solenopsin alkaloids extracted from two species of fire ants against the protozoan parasite *Trypanosoma cruzi*, the aetiologic agent of Chagas disease. Although IC_50_ determinations showed that solenopsins are more toxic to the parasite than benznidazole, the drug of choice for Chagas disease treatment, the ant alkaloids presented a lower selectivity index. As a result of exposure to the alkaloids, the parasites became swollen and rounded in shape, with hypertrophied contractile vacuoles and intense cytoplasmic vacuolization, possibly resulting in osmotic stress; no accumulation of multiple kinetoplasts and/or nuclei was detected. Overexpressing phosphatidylinositol 3-kinase—an enzyme essential for osmoregulation that is a known target of solenopsins in mammalian cells—did not prevent swelling and vacuolization, nor did it counteract the toxic effects of alkaloids on the parasites. Additional experimental results suggested that solenopsins induced a type of autophagic and programmed cell death in *T. cruzi*. Solenopsins also reduced the intracellular proliferation of *T. cruzi* amastigotes in infected macrophages in a concentration-dependent manner and demonstrated activity against *Trypanosoma brucei rhodesiense* bloodstream forms, which is another important aetiological kinetoplastid parasite. The results suggest the potential of solenopsins as novel natural drugs against neglected parasitic diseases caused by kinetoplastids.

## Introduction

Alkaloids are organic compounds containing a nitrogen atom usually associated with the cyclic chain within its structure^1^. They are a large, diverse group of chemicals, in which some compounds are known as poisons (e.g., strychnine), stimulants (e.g., caffeine), and analgesics (e.g., morphine)^2^. Alkaloids are mostly found in plants but have also been identified from a number of animals, such as frogs and ants^1^. Recently, several studies have evaluated the potential of antimicrobial alkaloids as prospective new drugs against diseases caused by bacteria^3^ and protozoan parasites^4^. Some alkaloids can pass the blood–brain barrier, such as cinchona, ergotamine, and alkaloids bearing a tertiary amine^5^.

Solenopsins are a recent, heterogeneous group of alkaloids typically yielding aliphatic chains linked to a piperidine ring^6,7^, originally isolated from fire ants. Solenopsins possess a few biological activities, including antibacterial^8,9^, antibiofilm^10,11^ and fungistatic^12^ activity. Biochemically, solenopsins can interfere in several pathways, such as quorum-sensing factors^11^, neuromuscular transmission^13^, and histamine release from mast cells^14^, and can serve as ATPases^15,16^ and sodium-pump inhibitors^17^. Among other activities^18,19^, solenopsins can inhibit phosphatidylinositol 3-kinase signalling and angiogenesis^20,21^, which is within the scope of the present investigation.

Chagas disease, aka American trypanosomiasis, is a potentially life-threatening disorder caused by different strains of the protozoan *Trypanosoma cruzi*, currently affecting approximately 8 million individuals worldwide (and estimated 25 million people are at risk), leading to over 10,000 deaths per year^22^. Two nitrogenous drugs are employed to treat Chagas disease: nifurtimox and benznidazole. Although markedly toxic to humans, these compounds are useful when administered during the acute phase of the disease, leading to a ca. 80% cure rate, but they prove generally ineffective during the chronic phase of the disease^22,23^. Therefore, there is an urgent need for more effective therapies targeting the chronic phase of Chagas disease. Some other molecules have been proposed, currently pending the necessary clinical steps^24–26^ prior to field applications. Among the few alkaloids proposed, the most promising was bisbenzylisoquinoline from the plant *Albertisia papuana* named daphnoline, which yielded up to a 70% parasitological cure in chronically infected mice^27^. To our knowledge, studies concerning the activity of alkaloids from animal venoms against protozoan parasites have never been performed.

In the present study, we tested the effects of piperidine alkaloids known as solenopsins against different life forms of *T. cruzi*. The alkaloids were extracted from the venom of the invasive fire ants *Solenopsis invicta* and *Solenopsis saevissima*. The protozoans were lab-cultured and observed for morphological and biochemical changes following exposure to alkaloids in the different lifecycle stages epimastigotes (i.e., replicative non-infecting forms from the insect vector) and amastigotes (i.e., the replicative infective form from inside mammalian cells).

## Results

### The solenopsins isolated from the venom of the fire ants *S. invicta* and *S. saevissima*

**Figure 1:**
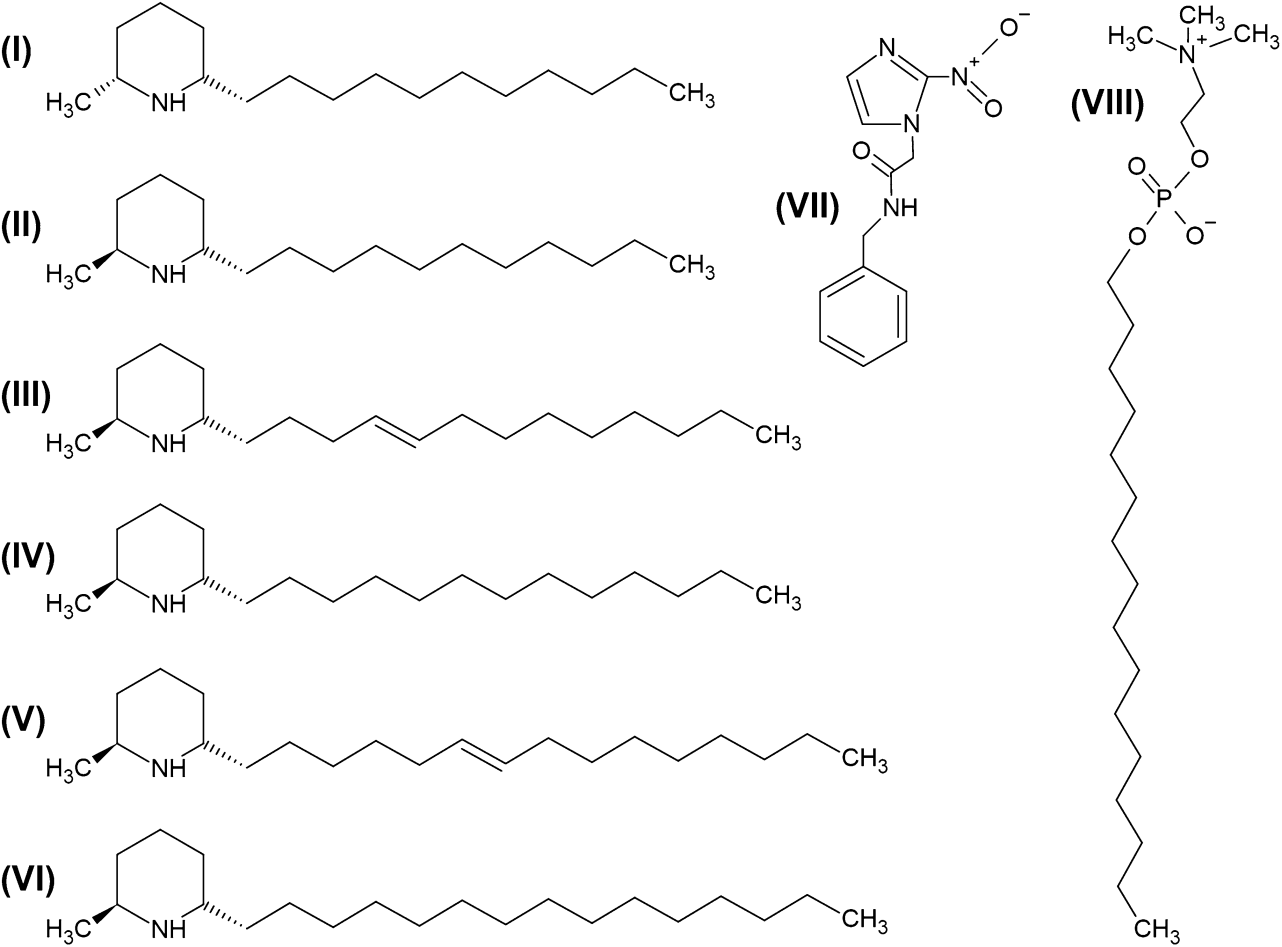
Chemical structure of all tested compounds used in the present study. Chemical structure of the solenopsins (**I**–**VI**), benznidazole (**VII**) and miltefosine (**VIII**). Additional information about the solenopsin alkaloids I–VI and their relative abundance in the venom of *Solenopsis invicta* and *S. saevissima* can be found in Table 1.

After extraction and fractionation, the composition of the solenopsins from the venom of *S. invicta* and *S. saevissima* was assessed by gas chromatography (GC–MS). Total ion chromatograms (Fig. S1) illustrate the diversity of solenopsin analogues found in the venom of each fire ant species, and their chemical structures and distribution (compounds I–VI) are presented in Fig. 1 and Table 1, respectively. By averaging the approximate composition analyses of Table 1, we estimated the approximate molecular masses of 288 g mol^−1^ and 253 g mol^−1^ for solenopsin extracts from *S. invicta* and *S. saevissima*, respectively.

**Table 1:** Identification and relative distribution of the solenopsins purified from the venom of *Solenopsis invicta* and *Solenopsis saevissima*.

Alkaloid	Compound	Short name	M^+^ (*m/z*)^a^	*Solenopsis invicta*	*Solenopsis saevissima*
(I) Isosolenopsin A	*cis*-2-Me-6-undecyl piperidine	*cis*-C11	253	n.d.^b^	8%
(II) Solenopsin A	*trans*-2-Me-6-undecyl piperidine	*trans*-C11	253	2%	89%
(III) Dehydrosolenopsin B	*trans*-2-Me-6-tridecenyl piperidine	*trans*-C13:1	279	54%	1%
(IV) Solenopsin B	*trans*-2-Me-6-tridecyl piperidine	*trans*-C13	281	10%	1%
(V) Dehydrosolenopsin C	*trans*-2-Me-6-pentadecenyl piperidine	*trans*-C15:1	307	25%	n.d
(VI) Solenopsin C	*trans*-2-Me-6-pentadecyl piperidine	*trans*-C15	309	8%	n.d

^a^Molecular ion (M^+^) identified by GC–MS.

^b^n.d. = not detected.

### Solenopsins inhibit proliferation but not alter epimastigotes of *T. cruzi*

To quantify the toxic effect of solenopsins on the parasites, we tested their effects against the proliferation of *T. cruzi* epimastigote forms of two different strains: Dm-28c and CL-Brener. As summarised in Table 2 (also Fig. S2A), after 48 h of incubation, solenopsins presented IC_50_ values against Dm-28c strain epimastigotes of 0.87 μM for *S. invicta* and 0.64 μM for *S. saevissima*, which are considerably lower than the IC_50_ values of the usual treatment drugs miltefosine (3.15 μM)^28^ (Fig. 1, compound VIII) and benznidazole (36.80 μM)^29,30^ (Fig. 1, compound VII). A similar range of IC_50_ values were also obtained against CL-Brener epimastigotes cultured in the presence of solenopsins of *S. invicta* (0.73 μM) and solenopsins of *S. saevissima* (0.58 μM; see Table 2; Fig. S2B). Parasites treated with either solenopsin extracts within the range of 0.25–0.5 × IC_50_ values for up to 8 days later recovered culture growth capacity when the solenopsins were removed (Fig. S2C), indicating that the replication inhibition induced by solenopsins is reversible.

**Table 2:** In vitro sensitivity tests using *Trypanossoma cruzi* and *Trypanosoma brucei rhodesiense* against solenopsin alkaloids of *Solenopsis invicta*, *Solenopsis saevissima*, Benznidazole and Miltefosine.

Tested chemicals	*T. cruzi* Epi Dm28c^a^	*T. cruzi* Epi CL-Brener^a^	*T. cruzi* Epi (PI3K) CL-Brener^a^	*T. cruzi* Ama Dm28c^a^	*T. brucei* BSF^a^	BMDM^b^	SI^c^ Epi Dm28c	SI^c^ Ama Dm28c	SI^c^ BSF
*S. invicta*	0.87 ± 0.63 (A)	0.73 ± 0.09 (A)	0.56 ± 0.13 (A)	2.59 ± 0.99 (A)	0.42 ± 0.03 (A)	5.29 ± 2.25 (A)	6.08	2.04	12.59
*S. saevissima*	0.64 ± 0.25 (A)	0.58 ± 0.12 (A)	0.57 ± 0.09 (A)	2.47 ± 0.48 (A)	n.a.^d^	8.77 ± 1.80 (A)	13.70	3.55	n.a.^d^
Benznidazole	36.80 ± 3.36 (B)	n.a.^d^	n.a.^d^	7.30 ± 1.17 (B)	n.a.^d^	822.9 ± 28.2 (B)	22.36	112.72	n.a.^d^
Miltefosine	3.15 ± 0.37 (C)	n.a.^d^	n.a.^d^	0.70 ± 0.20^e^	n.a.^d^	65.5 ± 4.8^e^	20.79	93.57	n.a.^d^

^a^IC_50_ and ^b^CC_50_ μM values from 3 independent experiments (*n* = 3) presented as means ± standard deviation (s.d.). Different capital letters in parenthesis within the same column indicate significant statistical difference by nonparametric Dunn's test posthoc to Kruskal–Wallis at alpha = 0.05.

^c^Selective Index (SI) = CC_50_ for BMDM / IC_50_ for parasite (Epi, Ama or BSF).

^d^n.a. = not assigned.

^e^IC_50_ μM values of Miltefosine for Ama Dm28c from Saraiva et al.^28^ and of Miltefosine for BMDM from Glaser et al.^90^.

**Figure 2:**
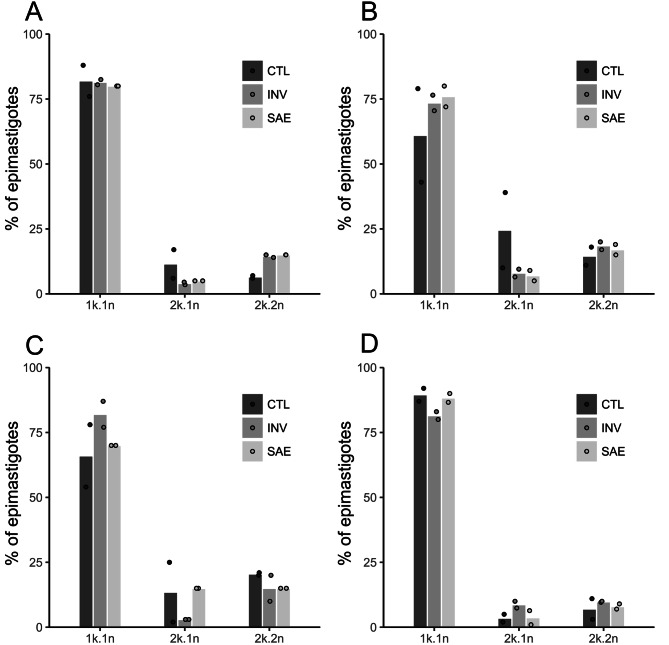
Effects of solenopsins in the cell-cycle of axenically grown epimastigote forms of *Trypanosoma cruzi*. Giemsa stained epimastigotes (500 per each day) obtained from cultures maintained in absence (CTL, dark grey bars) or presence of 0.3 µM solenopsins from *S. invicta* (INV, grey bars) and *S. saevissima* (SAE, light gray bars), respectively, were evaluated at days 1 (**A**), 3 (**B**), 5 (**C**), and 7 (**D**) for the presence of 1 kinetoplast and 1 nucleus (1k/1n), 2 kinetoplasts and 1 nucleus (2k/1n), and 2 kinetoplasts and 2 nuclei (2k/2n). Bars represent the mean % of two independent experiments, and dots are measured data points.

To quantify the possible effect of solenopsins on the cell cycle, Giemsa-stained epimastigotes were surveyed for the development of multiple nuclei (n) and kinetoplasts (k) over time. A total of 500 epimastigotes were evaluated by light microscopy over 7 days of culture (Fig. 2). As epimastigote forms cannot be cultured in precise synchrony, an arbitrary zero time-point was set when > 95% cells presented 1k and 1n. Following a stabilisation period, observed cultures underwent division cycles until no observable significant difference existed between treated and untreated parasites (days 1–3, Fig. 2A, B). Following 5–7 days of culture, there was still no observable difference between controls and treated cells in terms of 1k/1n, 2k/1n or 2k/2n proportions (Fig. 2C, D). Fluorescent intercalator displacement assays (Fig. S3) indicated that solenopsins are unable to intercalate in DNA, unlike berberine and emetine^31,32^ alkaloids, indicating that the activity to epimastigotes is likely not linked to direct DNA interaction.

### Solenopsins affect the morphology and long chain polyphosphate levels of *T. cruzi* epimastigotes

**Figure 3:**
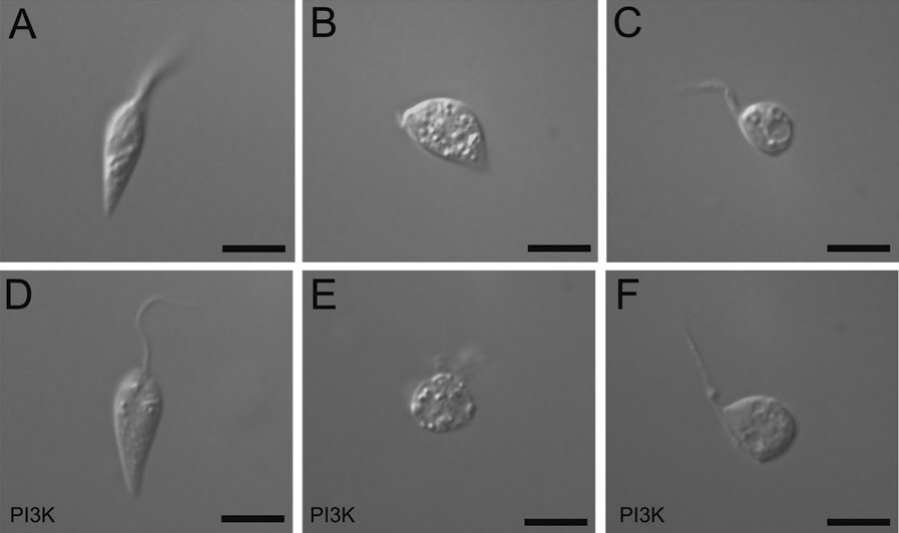
Nomarsky differential interferential light microscopy of *Trypanosoma cruzi* epimastigote forms incubated in the absence or presence of solenopsins. Cultures of wild type (**A**–**C**) or overexpressing PI3K (**D**–**F**) CL-Brener epimastigotes maintained for 48 h in the absence (**A**, **D**) or presence of 0.3 µM of solenopsins from *S. invicta* (**B**, **E**) and *S. saevissima* (**C**, **F**), respectively, were collected, washed in PBS and processed for observations in light microscopy as detailed in M&M. Bars = 10 µm.

The inhibition of *T. cruzi* epimastigote replication by solenopsins was followed by a cumulative concentration-dependent increase in aberrant rounded morphology (Fig. 3). While untreated cells maintained normal, fusiform shape (Fig. 3A), the treated epimastigotes showed an increase in the density of cytoplasmic vacuoles associated with altered rounded shapes, typical of cells undergoing osmotic stress, autophagy, and/or apoptosis (Fig. 3B, C). No significant increase in bi- or multi-flagellate forms was observed.

**Figure 4:**
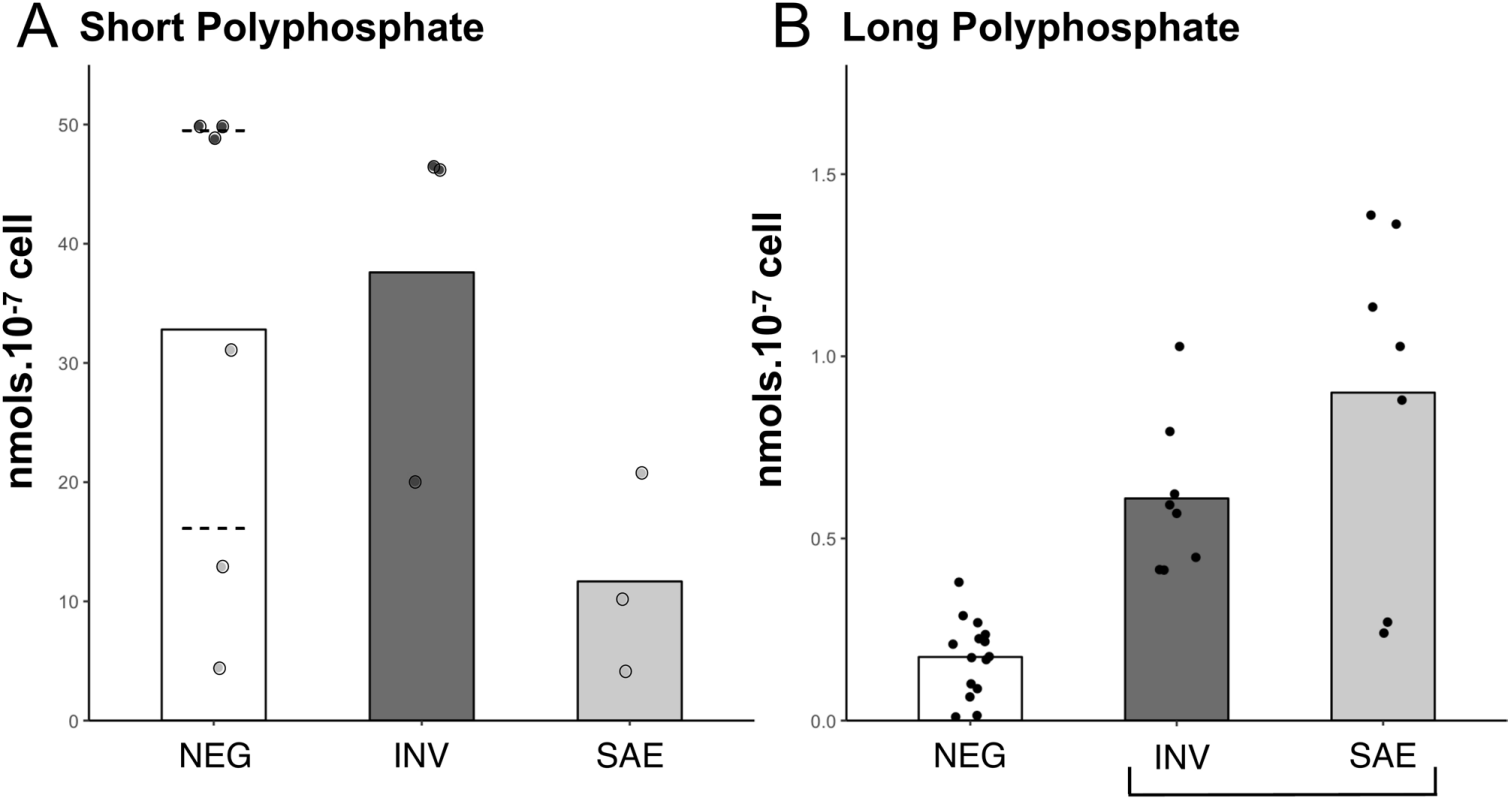
Effects of solenopsins in the polyphosphate chains accumulated by epimastigotes forms of *Trypanosoma cruzi*. After cultivation for 48 h in the absence (NEG) or presence of 0.3 µM of solenopsins from *Solenopsis invicta* (INV) or *S. saevissima* (SAE), CL-Brener epimastigotes were collected and the short chain (**A**) and long chain (**B**) polyphosphates (PolyP) were extracted and quantified as described in Methods. Bar plots indicate mean values where dots are the raw data (nmoles.10^–7^ parasites) of three independent experiments. Statistics: Results were compared by non-parametric Kruskal–Wallis, and treatments are grouped by statistical similarity at alpha = 0.05 where indicated with brackets. For details refer to supplementary R script file.

It is known that polyphosphate (polyP) concentration changes drastically during the lifecycle of *T. cruzi*, especially when parasites are exposed to osmotic or alkaline stress^33,34^. Therefore, we compared polyP levels among treated and untreated parasites (Fig. 4) and observed that solenopsin-treated parasites consistently showed increased long-chain polyP concentrations but not short-chain polyP concentrations (Fig. 4A, B).

It has been reported that the enzyme *T. cruzi* TcVps34—a homologue of phosphatidylinositol 3-kinase (PI3K)—is actively involved in the recovery of parasites submitted to hypo-osmotic stress^35^ and participates in the regulation of autophagy^36^. However, it has been previously shown that solenopsins suppress PI3K activation^21^. We therefore hypothesised that solenopsins might similarly induce osmotic stress and/or autophagy in epimastigotes by inhibiting TcVps34. To test this hypothesis, we compared the growth curves of wild-type and CL-Brener epimastigotes overexpressing TcVps34 (TcPI3K) and exposed to different solenopsin concentrations. TcPI3K overexpression did not affect the IC_50_ of solenopsins from *S. invicta* (0.56 μM) and *S. saevissima* (0.57 μM; Table 2; Fig. S1B). TcPI3K overexpression typically leads to enlarged contractile vacuoles and hypertrophic alterations near the cytostome and flagellar pocket of epimastigotes^35^ (see Fig. 3D). Nonetheless, TcPI3K overexpression did not prevent solenopsin-induced parasite rounding and vacuolization (Fig. 3E, F) or the accumulation of long-chain polyP (not shown).

**Figure 5:**
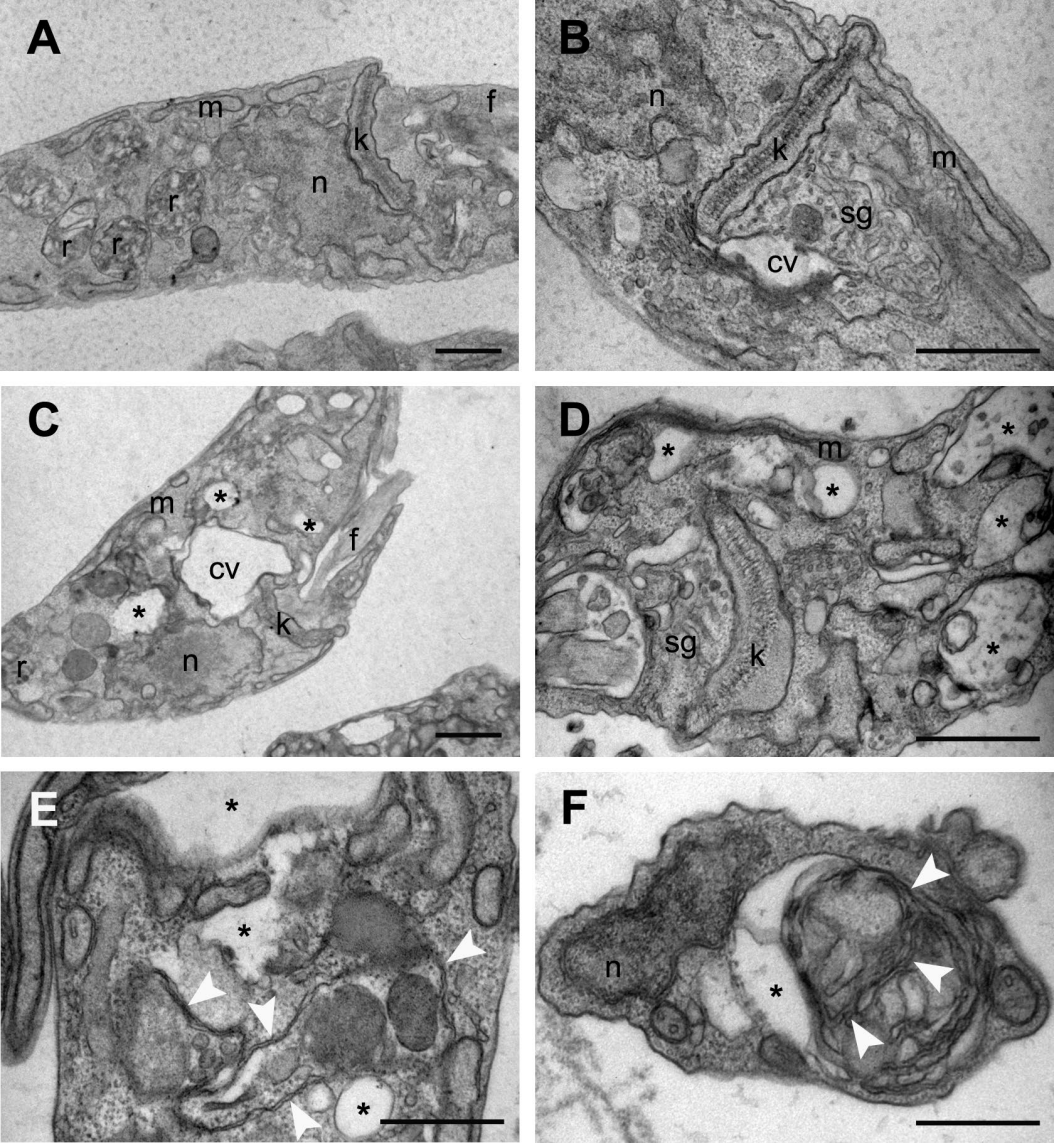
Transmission electron microscopy of *Trypanossoma cruzi* epimastigotes incubated in the absence or presence of solenopsins. Epimastigotes of the CL-Brener strain were incubated for 48 h in BHI–FCS medium in the absence (**A**, **B**) or presence of 0.3 µM solenopsins (**C**–**F**). The parasites were processed and observed by transmission electron microscopy as described in M&M. (**A**, **B**) Control untreated parasites showing the nucleus (n), kinetoplast (k), reservosomes (r), mitochondrion (m) flagellum (f) and tubular structures with vesicles which presumably correspond to the spongiome (sg) surrounding a space that can be part of the contractile vacuole (cv) bladder. (**C**–**F**) Epimastigotes after treatment with solenopsins showed intense vacuolization of the cytoplasm (*) and an eventual hyperthrophic contractile vacuole (cv) but no mitochondrion swelling (m). Cells also presented vacuolar structures displaying complex double membrane invaginations or vesicles that resemble autophagosomal structures (white arrow heads). Bars = 0.5 µm.

The morphological alterations induced by solenopsin exposure were further analysed by transmission electron microscopy (Fig. 5) using cells with regular morphology (Fig. 5A, B), indicating the onset of vacuolization (asterisks in Fig. 5C–F) and hypertrophied vacuoles (Fig. 5E, F) in solenopsin-treated parasites. No alterations in the morphology of mitochondria or nuclei were observed (Fig. 5C, D). In addition, solenopsin-treated epimastigotes also presented vacuolar structures displaying complex double-membrane invaginations and vesicles resembling autophagosomes (Fig. 5E, F, white arrowheads). Similar ultrastructural alterations were observed in solenopsin-treated epimastigotes overexpressing PI3K (not shown).

### Solenopsins induce autophagy and programmed incidental cell death in *T. cruzi* epimastigotes

**Figure 6:**
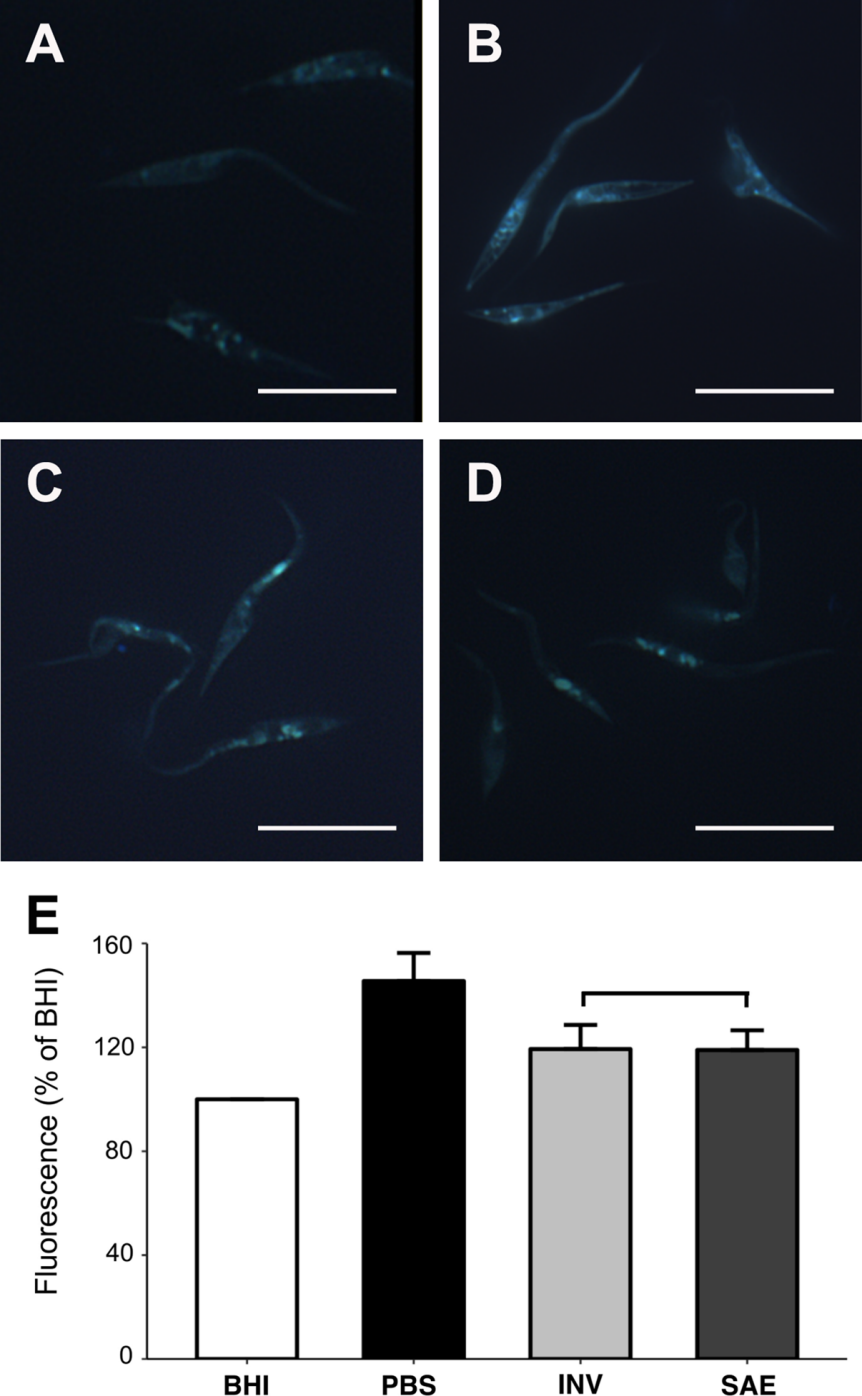
Labelling of *Trypanossoma cruzi* epimastigotes with monodansylcadaverine (MDC) after treatment in the absence or presence of solenopsins. After cultivation for 48 h in BHI–FCS medium (**A**, BHI), PBS (**B**, PBS), or BHI–FCS medium supplemented with 0.3 µM of solenopsins from *Solenopsis invicta* (**C**, INV) or *S. saevissima* (**D**, SAE), CL-Brener epimastigotes were collected and incubated with 50 μM of MDC for 1 h at 28 °C, washed 3 × in PBS, fixed, and examined in an epifluorescence microscope as described in Methods. Bars = 20 µm. To quantify the MDC labelling, 5 × 10^7^ parasites treated as described were lysed in Tris-HCl (pH 8.0) with 1% SDS, centrifuged, and fluorescence from the supernatants measured with a microplate reader. Plot (**E**) presents the mean ± SD fluorescence values (in % relative to BHI) of three independent experiments. Statistics: treatments were compared with Kruskal–Wallis at alpha = 0.05; INV (light-grey bar) and SAE (dark-grey bar) indicates statistically similar treatment results; an asterisk indicates values statistically different from the control (BHI). For details on analyses (e.g. *p* values), see the supplementary R script file.

Solenopsin-treated epimastigotes presented increased numbers of cytoplasmic vacuoles and cytosolic concentric double-membrane inclusions symptomatic of autophagy and apoptosis (Fig. 5). We searched for evidence of autophagy by incubating cells with monodansylcadaverine (MDC), a marker of autophagic vacuoles in vivo^37^ (Fig. 6). Epimastigotes show more intensive MDC staining when incubated under low-nutrient conditions (Fig. 6B, E) than under control conditions (Fig. 6A, E); similar intense MCD labelling was observed in solenopsin-treated parasites (Fig. 6C–E).

**Figure 7:**
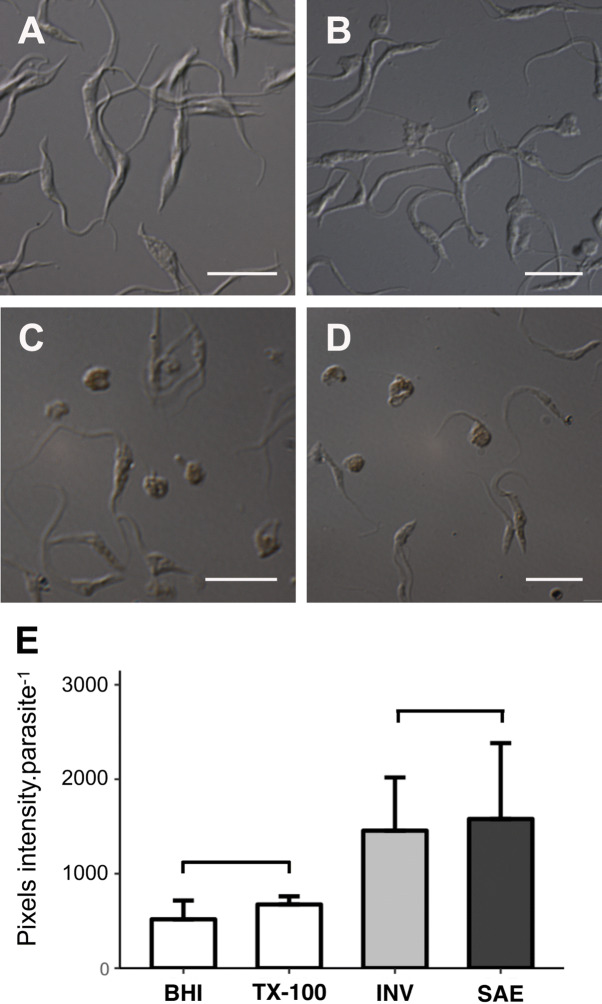
Detection of free DNA 3′-OH ends after incubation of *Trypanossoma cruzi* epimastigotes in the absence or presence of solenopsins. After cultivation for 24 h in BHI–FCS medium alone (**A**, BHI) or supplemented with either 0.07% TX-100 (**B**, TX-100) or 2.5 µM of solenopsins from *Solenopsis invicta* (**C**, INV) or *S. saevissima* (**D**, SAE), CL-Brener epimastigotes were collected, washed (3 ×) in PBS, suspended in PBS (around 2 × 10^7^ cells mL^−1^), allowed to adhered to a poly-l-lysine embedded slide, processed through ApopTag technology, and observed under a light microscope. The obtained differential interference contrast images enable the comparison of unstained parasites with normal morphology (**A**), parasites with altered morphology that do not have any colour (**B**) that is compatible with necrosis induction, and stained parasites that also have altered morphology (**C**, **D**) that is compatible with the induction of apoptosis. Bars = 10 μm. The graphics (**E**) represent the mean ± SD of pixels intensity for parasites for each treatment: BHI (white bar), TX-100 (dark bar), INV (light-grey bar), SAE (dark-grey bar) from three independent experiments. Statistics: treatments were compared with Kruskal–Wallis at alpha = 0.05; the BHI/TX-100 and INV/SAE bars indicate statistically similar treatment results; an asterisk indicates values statistically different from controls. For details on analyses (e.g. *p* values), see the supplementary R script file.

The capacity of solenopsins to induce programmed cell death was also analysed by measuring DNA nicks and fragmentation (Fig. 7). Relative to untreated controls (Fig. 7A) and cells necrotized with Triton X-100 (Fig. 7B), solenopsin-treated epimastigotes stained positively for DNA 3′-OH free ends (Fig. 7D); the difference was statistically significant (Fig. 7E). Taken together, these results strongly suggest that solenopsins induce autophagy and incidental cell death in epimastigote forms of *T. cruzi*^38,39^.

### Solenopsins are trypanocidal to peritoneal *T. cruzi-infected* macrophages and toxic to *T. brucei rhodesiense*

**Figure 8:**
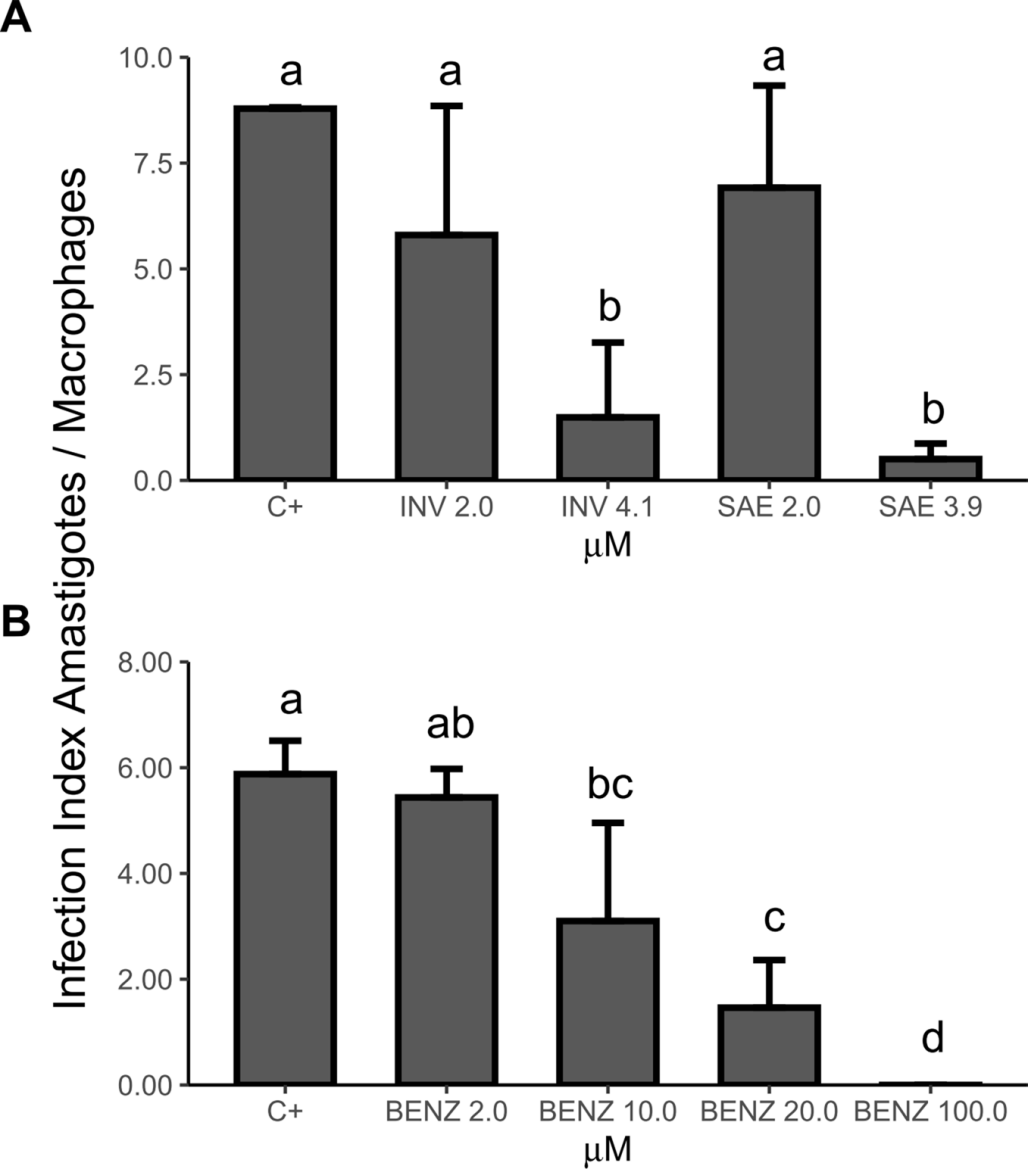
Effects of the solenopsins on macrophages infected with *Trypanossoma cruzi*. Mouse peritoneal macrophages plated on glass cover slips in 12-well plates were incubated with tissue culture trypomastigotes (TCTs) at a multiplicity of infection of 3 parasites per cell. After 12 h, wells were washed 5 × and the cultures were incubated in the absence (C+) or presence of solenopsins from *Solenopsis invicta* (INV) and *S. saevissima* (SAE) (**A**) or benznidazole (BENZ) (**B**) as indicated in the bottom of each graph. After 48 h, the cover slips were washed, and the cells were fixed and stained with InstantProv haematological stain. The infection index based in the percentage of infected macrophages and the number of amastigotes per infected macrophage was estimated by direct counting of ≥ 300 fields. Bar plots are means and different letters on the bars indicate significant difference between the treatments by nonparametric Dunn's test posthoc to Kruskal–Wallis at alpha = 0.05 (*n* = 2). For details on analyses (e.g. *p* values), see the supplementary R script file.

The activity of the solenopsins was assessed against cultured mammalian BMDM, CHO, and LLC-MK_2_ cell lines using 3-(4,5-dimethyl-2-thiazolyl)-2,5-diphenyl-2H-tetrazoliumbromide (MTT)^40^ and lactate dehydrogenase (LDH)^41^ assays. Solenopsins proved toxic to CHO cells at concentrations ≥ 7.5 μM (Fig. S4A–B) based on MTT and LDH assay methods. Solenopsins were more toxic to primary bone marrow-derived macrophages (BMDMs) than to other cell lineages, possibly as a result of the immortalized phenotype of lineages (Fig. S4C). The effects of solenopsins on *T. cruzi*-infected murine peritoneal macrophages were evaluated in vitro under similar settings and quantified as the infectivity index (i.e., number of infected macrophages x number of intracellular amastigotes/total number of macrophages) following staining and counting (Fig. 8). Solenopsins reduced the number of infected macrophages and the number of amastigotes inside infected macrophages (Fig. 8A) in a concentration-dependent manner, yielding an estimated IC_50_ value of 2.59 µM for *S. invicta* solenopsins and 2.47 μM for *S. saevissima* solenopsins (Table 2), which was significantly lower than the IC_50_ of benznidazole (Fig. 8B) with a value of 7.30 μM (Table 2). However, based on the toxic effects observed against BMDM cells, the selective index values of the solenopsin alkaloids for intracellular amastigote forms (2–3.5) were significantly lower than those observed for the reference compounds benznidazole (112.7) or miltefosine (93.5) (Table 2). Solenopsins also proved toxic against *T. brucei rhodesiense* (Figure S5), the aetiological agent of human sleeping sickness. Solenopsins from *S. invicta* inhibited the growth of *T. brucei rhodesiense* (Fig. S5) bloodstream forms in a concentration-dependent manner, yielding an estimated IC_50_ value of 0.42 µM (Table 2).

## Discussion

Arthropod venoms are a rich, essentially untapped source for bioactive molecules. Among venomous insects, ants are remarkably chemically diverse^42^, as illustrated by a recent increase in the number of studies surveying the biomedical applications of ant toxins, such as the Brazilian giant ant *Dinoponera quadriceps*^43^. Among several other biomedical activities, the venom peptides of *D. quadriceps* were reported to be toxic to *T. cruzi*^44^. Notwithstanding, the venom alkaloids of ants have remained untested against trypanosomatids.

The fire ant alkaloids known as solenopsins abound in the venom of these ants following typical species-specific configurations^45,46^. The two fire ant species selected for this study represent extremes in solenopsin chemical diversity; *S. invicta* venom includes almost all known solenopsin analogues (Fig. 1, compounds II–VI), and the cryptic species of *S. saevissima* abounds in analogues of solenopsin A^45^ (Fig. 1, compounds I–II), which is to date, the best-studied solenopsin alkaloid^18^. Despite conspicuous differences in the solenopsin analogue proportions isolated from both species (Table 1), similar IC_50_ growth inhibition values were obtained against the *T. cruzi* epimastigote and amastigote forms (Table 2). This contrasts with the fact that different solenopsins exhibit a diverse cytotoxic effect on different bacterial species^9,18^, suggesting that different mixtures of solenopsin analogues have similar effects against *T. cruzi*. A number of piperidine alkaloids can pass the blood brain barrier, such as nicotine^4^. Such properties, if present in solenopsins, could prove useful against some protozoan infections, e.g., in the brain, such as sleeping sickness caused by *Trypanosoma brucei* species^47^, and where treatment is impaired by a low tissue penetration by current medications, e.g., in leishmaniasis caused by *Leishmania* species^48^ or in Chagas disease caused by *T. cruzi*^49^.

The negative effects of solenopsins on the proliferation of *T. cruzi* epimastigote forms were reversible, similar to previous reports on other alkaloids^50–54^. Additionally, the observation that epimastigotes become rounded with increased intracellular vacuolization and complex membrane invaginations has been reported for most studies involving the treatment of *T. cruzi* with alkaloids. A remarkable difference herein, however, was the absence of a clear swelling of the mitochondrial matrix, as observed with camptothecin^48,50^ or piperine^52^. Additionally, solenopsins delayed cell cycle progression but did not block the synthesis or segregation of nuclear and kinetoplast DNA, as reported with Taxol^50^ or colchicine^54^. Finally, unlike the effects caused by vinblastine and vincristine that induce cytokinesis arrest in *T. cruzi* epimastigotes^51^, exposure to solenopsins did not induce the formation of multinucleate cells.

Either the molecular interactions of solenopsins with cellular components are reversible, or parasites can somehow compensate for the toxic effects. The growth inhibition caused by solenopsins—with no arrest at any specific cell cycle stage—might be associated with the inhibition of the total synthesis of macromolecules, indirectly leading to a larger G_0_ “stationary” phase. In fact, the cell cycle is extremely sensitive to any kind of chemical or physical stress, due to several control mechanisms that ensure the capacity for progression to the next phase^55^. Therefore, several environmental stresses can generate a delay in cell cycle progression of different cells.

Antimicrobial alkaloids are typically tested for DNA intercalation^56,57^. The fluorescent DNA intercalator assay suggested that solenopsins either present weak DNA interactions, e.g., weak ionic interactions, or merely bind to DNA-associated histones or enzymes. Concerning histone interactions, it has been shown that the overexpression of histone (H1) in *Leishmania* generates a delay in the cell cycle from increased histone interactions with nuclear DNA^58^. Chaetocin, a fungal toxin possessing an epipolythiodioxopiperazine alkaloid moiety and a nonspecific inhibitor of histone lysine methyltransferases^59,60^, has been shown to impair proliferation, arrest cell cycle progression and induce nucleolar disassembly in *T. cruzi*^53^. The inhibition of other cell functions could disrupt the cell cycle and morphology, as illustrated by the alkaloid vinblastine impairing transcriptional and post-transcriptional regulatory levels and regulating tubulin expression in *T. cruzi*^54^. Similar effects could also result from solenopsin alkaloids affecting membrane transporters, as has been already described in mammalian cells^13,17,18^. Nevertheless, the observations reported here delineate the scope of the mechanisms of action of solenopsins acting on *T. cruzi*.

Previous studies have shown that solenopsins inhibit ATPases in mammals^15^, Na^+^–K^+^ ATPase activity in chickens and catfish^16^, and suppress PI3K activation and/or associated downstream phosphorylation in mammals—for example, protein kinase B (PKB/Akt) and its substrate forkhead box 01a (FOXO1A)^21^. The functional inhibition of Akt activity has been linked to reduced 3-phosphoinositide-dependent protein kinase 1 (PDK1) activation and increased mitochondrial reactive oxygen species (ROS) and autophagosome formation, lethal to several malignant tumour cell lines^19^.

Solenopsins share the long alkyl side chains observed in ether-phospholipids, and sphingolipids have a structural resemblance to miltefosine (Fig. 1, compound VIII), edelfosine, perifosine^21^ and ceramide^19,20^ in that both have a positively charged amine moiety. Indeed, like solenopsins, ether-lipid analogues interact with Akt and on other potential alternative signalling targets, such as mitogen-activated protein kinase (MAPK) and protein kinase C (PKC) in cancer cells^61^. Miltefosine has been used to treat leishmaniasis in humans^62^ and has already been shown to be toxic against *T. cruzi* both in vitro and in vivo^28^. Based on the present ultrastructural observations, solenopsins presented similar effects previously ascribed to ether-lipid analogues, as illustrated by autophagy via autophagosome formation, the onset of membranes around organelles and cytosolic structures, and apoptosis-like cell death resulting in DNA fragmentation and formation of apoptotic bodies^63^. At the molecular level, despite the intrinsic pro-inflammatory effects^28^, the main targets of miltefosine are (1) the initial biosynthesis enzymes of ether lipids involved in the synthesis of glycosylphosphatidylinositol anchors^64^, (2) membrane Na^+^-ATPases^65^, and (3) PKC^65^. Notwithstanding such hypothetical structure–function correlations between solenopsins and ether-lipid analogues, additional studies are still needed to elucidate the pathways targeted by solenopsins in *T. cruzi*.

As mentioned, solenopsin-treated parasites typically become rounded, with multiple cytoplasmic vacuoles (Figs. 3, 5). Several adverse environmental conditions (e.g., osmotic stress) can induce cell rounding in *T. cruzi* epimastigotes, and some altered molecular targets have been associated with this morphological alteration, such as protein phosphatase type 1^66^. Treatment of *T. cruzi* epimastigotes with calyculin A—an inhibitor of type 1 phosphatases—can induce cell rounding and arrest the cell cycle, but it has not been clarified whether these effects are derived from phosphatase inhibition^66^. An anterior hypertrophied vacuole close to the flagellar pocket was observed in solenopsin-treated parasites (Fig. 5), which could be a response to hypo-osmotic stress. The same contractile vacuole is considered essential to the parasite cell cycle, as it is exposed to drastic environmental alterations (involving osmotic and pH stress) in switching between different hosts^37,67^. However, this interpretation that solenopsins cause osmotic imbalance was not in principle supported by the observed greater susceptibility of the CL-Brener strain, as it overexpresses PI3K that should provide augmented adaptability to osmotic stress.

Cellular polyP levels are known to vary in *T. cruzi* with stress and are associated with a reduction in polymeric phosphate levels^67,68^. Exposure to solenopsins increased long-chain polyP levels; however, no alterations in the levels of short chain polymers were observed. This altered polyP chain phenotype had not yet been previously described for *T. cruzi*, even when epimastigotes were subjected to different stresses^30^. This result further argues against an activity mechanism of solenopsins in which the compounds act as osmotic stress inducers (e.g., via membrane interaction). A direct interaction of the alkaloids with the factors regulating polyP levels cannot, however, be ruled out yet. The altered proportions between the short and long polyP levels induced by solenopsin exposure could be associated with diverse pathways. For instance, it is believed that polyP short chains are preferentially consumed in energy metabolism through the cleavage of phosphodiester bonds, leaving long-chain polyphosphates available to regulatory functions^69^. Several studies with bacteria and fungi suggested that long-chain polyP plays an important role in the response to stress, such as in bacteria surviving under very low nutrient availability^70^. In this scenario, the accumulation of polyP during the logarithmic growth phase seems central to bacterial survival, but this accumulation normally does not take place at the same phase in *T. cruzi*^36^. We therefore interpret the increased long-chain polyP levels as a physiological stress response to exposure to solenopsins.

The morphological changes observed in solenopsin-treated parasites are typical of autophagy (cytoplasmic vacuolization and increased MDC binding) and cell death (i.e., retracted cytoplasm and fragmented DNA). Similar effects were reported on protists^71,72^, including *T. cruzi*^39,73–76^ and *T. brucei*^77^ exposed to other alkaloids like piperidine analogues. The collected evidence indicates that solenopsins act on interconnected pathways. Therefore, it seems worthwhile to explore whether ROS and Ca^+2^ levels (e.g., in the mitochondria) are also affected by exposure to solenopsins and would further indicate whether the alkaloids can mediate *T. cruzi* apoptosis^54,76^.

We have demonstrated that solenopsins are also active against the intracellular infectious amastigote *T. cruzi.* The obtained IC_50_ values that induced a reduction in the number of intracellular amastigotes were approximately 2.5 µM for the different solenopsin extracts, which is considerably lower than the values reported for other tested compounds, such as benznidazole (Table 2), piperine^52^ and naphtoquinolones^63^. These values are also lower than the drug concentrations that suppress PI3K activation in SVR murine endothelial cells^19^ or the concentrations used to reduce respiration and increase reactive oxygen species to kill cancerous cell lines or even to elevate Akt phosphorylation^19^ as a biochemical barrier restoration agent to enhance inflammation in mice challenged with psoriasis^20^. The solenopsins isolated from *S. saevissima* proved slightly stronger against amastigote *T. cruzi* than the solenopsins from *S. invicta,* perhaps as a result of greater macrophage penetration because of the shorter alkyl chain length. This is an interesting hypothesis pending further experimentation.

In short, solenopsin alkaloids proved toxic to *T. cruzi* amastigotes and epimastigotes, inducing morphological and biochemical alterations in the latter. This was congruent with pilot tests against *T. brucei rhodesiense*. Taken together, the results suggest that solenopsin alkaloids could be used in designing novel treatments against *T. cruzi* and other kinetoplastids that cause neglected parasitic diseases in humans. Although toxic at higher concentrations, synthetic solenopsins might be used candidates to increase treatment effectiveness and to decrease treatment toxicity in mammals, as illustrated by strategies adopted with other antibacterial compounds^78^.

## Materials and methods

### Cells and parasites

Epithelial cell lines LLC-MK_2_ (isolated from kidney of *Rhesus* monkey, *Macaca mulatta*—ATCC CCL-7, Rockville, MD, USA) and CHO (from ovaries of the Chinese hamster, *Cricetulus griseus*—ATCC CCL-61, Rio de Janeiro Cell Bank) were routinely maintained in RPMI 1640 (Sigma-Aldrich, USA) and DMEM media (Sigma-Aldrich, USA), respectively, both containing 5% (v/v) heat-inactivated fetal calf serum (FCS, Gibco, USA), 2 mM l-glutamine (Sigma-Aldrich, USA), 100 µg mL^−1^ streptomycin (Gibco, USA) and 100 IU mL^−1^ penicillin (Gibco, USA). Cells were incubated in a humidified incubator at 37 °C and 5% CO_2_ atmosphere. Every 48 h approximately, confluent cell layers were dissociated using 0.005% (v/v) trypsin–EDTA solution (Sigma-Aldrich, USA) diluted in phosphate buffered saline (PBS buffer, 150 mM NaCl, 45.84 mM NaH_2_PO_4_ and 9.5 mM Na_2_HPO_4_, pH 7.4), suspended in fresh medium, and transferred to new culture flasks. Bone marrow-derived macrophages (BMDMs) were produced from femoral bone marrow cells by culture for 7 days in DMEM supplemented with 20% L929 conditioned medium as described elsewhere^79,80^. To obtain mouse peritoneal macrophages, 4% thioglycollate solution (Sigma-Aldrich) was injected intraperitonially into C57BL/6 mice, and peritoneal cells were collected by chilled RPMI washes 4 days after injection to obtain the elicited macrophages. The protocol for animal use was approved by the Ethics Committee from Centro de Ciências da Saúde of UFRJ (number IBCCF-085) and all methods were performed in accordance with the relevant guidelines and regulations. The macrophages were seeded in a 12 well plate with coverslips in the bottom of each well and the adherent cells obtained after a period of 24 h were used for the infection experiments with tissue culture-derived *T. cruzi* trypomastigotes (TCTs) described further below.

Axenic cultures of CL-Brener and Dm-28c strains *T. cruzi* epimastigotes were obtained from the culture collection of Fundação Oswaldo Cruz (Fiocruz, Rio de Janeiro, Brazil). Parasites were routinely grown in complex medium containing 37 g L^−1^ Brain-Heart-Infusion (BHI) (Difco, USA), 10 µg mL^−1^ hemin (Sigma-Aldrich, USA), 20 µg mL^−1^ folic acid (Sigma, USA), 100 µg mL^−1^ streptomycin, 100 IU mL^−1^ penicillin and 5% (v/v) heat-inactivated FCS (BHI–FCS medium) in T25 culture flasks (Corning, USA) at 27 °C^81^. Cultures at stationary phase (0.5–1.0 × 10^8^ cells mL^−1^) were split 1:10 in T25 flasks every 5–7 days. Genetically modified parasites overexpressing TcVps34 (PI3K)^35^ were maintained as above, but in the presence of 500 µg mL^−1^ geneticin (US Biologicals, USA). Tissue culture-derived trypomastigotes (TCTs) were obtained from infected LLCMK_2_ cells as described elsewhere^81^, and were used here to infect murine peritoneal macrophages as described below.

In vitro cultures of the bloodstream forms of *T. brucei rhodesiense* were performed in HMI9 supplemented with 10% FCS and 10% serum plus (Gibco, USA)^82^.

### Extraction, purification and characterization of solenopsin alkaloids

Colonies of the fire ants *S. invicta* and *S. saevissima* were identified and venom alkaloids extracted as described previously^83^. In brief, fire ants’ mounds were collected at Ilha do Fundão, Rio de Janeiro, Brazil, separated from soil by slow flooding, and extracted with hexane (Merck Brasil, Brazil). The organic extract was further purified with hexane–acetone silica column (Sigma-Aldrich, USA)^83^. After solvents evaporation, relative proportion of solenopsin analogues was determined by gas chromatography-mass spectrometry (GC–MS) with a Shimadzu GCMS-QP2010 plus system using a fused silica RTX-5MS column (30 m, ID = 0.25 mm, d_r_ = 0.25 µm) (Restek, Bellefonte, PA, USA)^48^. Mass spectra were obtained using electron impact (EI) at 70 eV. The alkaloids were identified based on comparison with published GC profiles and mass spectra^6,84^. The alkaloids were further quantified by GC–MS using spiked known amounts of the methylxantine alkaloid caffeine (Sigma-Aldrich, USA) as an internal standard. Sample quantification was determined by dividing the total peak area of each alkaloid by the peak area of the internal standard, multiplied by the amount of standard added to the sample.

### Effects of solenopsin on the viability of mammalian cells

Cytotoxicity of solenopsins was evaluated against CHO and BMDM cells by the MTT assay^39^ and by the lactate dehydrogenase (LDH) assay^40^. In the MTT assay, cell viability was quantified by the ability of living cells to reduce the yellow dye 3-(4,5-dimethyl-2-thiazolyl)-2,5-diphenyl-2H-tetrazoliumbromide (MTT) to a purple Formazan product. The cells were plated in 24 well plates (7 × 10^4^ cells mL^−1^) and alkaloids dissolved in dimethyl sufoxide (DMSO; Pierce, USA) stock solution were tested in triplicate at 1:1 ascendant dilution (varying from 1 to 80 µM) in DMSO (final DMSO concentration was 0.1%). After 72 h of incubation, the supernatant was replaced by fresh medium containing MTT (0.5 mg mL^−1^) and 3 h later, the Formazan product was dissolved in DMSO and absorbance was measured at 595 nm in a DTX-880 spectrophotometer (Beckman Coulter, USA). In the LDH assay, cultures were incubated in 24 well plates for 72 h in the absence or presence of alkaloids as above, and the supernatants were collected (~ 900 µL), centrifuged at 5,000*g* for 10 min, and used for the LDH enzymatic test. Reactions were prepared in a 96 well plate containing 80 µL of supernatant and 120 µL of PBS buffer supplemented with 0.7 mM NADH (Bioexpress, USA) and 4.7 mM pyruvate (Adamas, USA). Changes in O.D. at 340 nm were quantified in a Spectra Max 250 micro plate reader (Molecular Devices, USA). The positive control was the supernatant of cell cultures kept for two hours in the presence of 0.1% of Triton X-100.

### Effects of solenopsins on the parasite’s proliferation in vitro

Parasites (5–20 × 10^4^ mL^−1^) were cultured in their respective medium for up to 16 days in the absence or presence of 0.1–384.0 µM of either the solenopsins or benznidazole (Lafepe, Brazil) from stock solutions kept in DMSO, or miltefosine (Cayman, USA) from stock solutions kept in PBS^28^. Numbers of parasites in each culture were estimated daily by direct counts in a Neubauer chamber, and their viability estimated by the Trypan blue exclusion method^33^. Control conditions were established with parasites cultured likewise but containing either PBS or with equivalent amounts of DMSO. The highest concentration of solvent used (0.1% DMSO) had no significant effect on the growth of the *T. cruzi* epimastigote forms or T. brucei rhodesiense bloodstream forms (not shown). The 50% inhibitory concentration (IC_50_) and its 95% confidence interval values were calculated plotting the inhibition (%) against the log of drug concentration fitted to a sigmoidal curve determined by non-linear regression^85^. Reversibility of the solenopsin effects on the growth of epimastigotes was evaluated by incubating the parasites with 0.16 µM and 0.30 μM of solenopsins, which are the approximate 0.25 × and 0.50 × 48 h IC_50_ values (described in "Results" section) of the alkaloids derived from *S. invicta* and *S. saevissima*, respectively. After incubation for up to 8 days in BHI–FResultsCS medium, the parasites were collected by centrifugation to 3,000*g* for 10 min at 4 °C, washed 2 × in PBS, suspended into fresh media free of solenopsins, and cultured for additional 8 days. Daily counts and viability evaluations of the parasites in the cultures were done as above.

### Effects of solenopsins on the proliferation of amastigote forms inside murine peritoneal macrophages

Murine peritoneal macrophages seeded onto round glass coverslips slides in 12 well plates were co-cultured with TCTs at a ratio of 1:3. After 12 h interaction, the macrophages were washed 5 × with 1 mL PBS to remove the non-internalized parasites and incubated in the absence or presence of solenopsin for 2 days as described above for the mammalian cell viability tests. After this period, the macrophages were fixed with methanol, stained with InstantProv hematological stain (NewProv, Brazil) and the slides observed under a light microscope. The infection index values (the number of infected macrophages X number of intracellular amastigotes/ total number of macrophages) were estimated by direct counting of at least 300 fields.

### Evaluation of the effects of solenopsins in the cell cycle and the morphology of epimastigote forms

Parasites were cultured in the absence or presence of 0.30 μM of solenopsins derived from either *S. saevissima* or *S. invicta* for up to 8 days as described above. Cell growth was estimated daily by Neubauer hemocytometer counts, and culture smears were Giemsa-stained^86^. At least 500 randomly-chosen microorganisms of each culture were evaluated and classified according to the number of kinetoplasts (k) and nuclei (n) per cell^35^. Observations and micrographs were taken in a Zeiss AxioPlan II light microscope using the AxioVision software coupled to an AxioCam MRC3 digital camera (Oberkochen, Germany). For observations using transmission electron microscopy (TEM), parasites were collected, washed three times in PBS and fixed in 2.5% (v/v) glutaraldehyde (Merck, Germany), 4.0% (w/v) formaldehyde (Merck, Germany), and 5 mM CaCl_2_ (Merck, Germany) in 0.1 M sodium cacodylate buffer pH 7.4 for 1 h at room temperature. After three washes in 0.1 M sodium cacodylate buffer pH 7.4, post fixation was carried out in 1.0% (w/v) osmium tetroxide (Merck, Germany), 0.8% (w/v) potassium ferrocyanide (Merck, Germany), 5 mM CaCl_2_ in 0.1 M sodium cacodylate buffer pH 7.4 for 1 h in the dark. Samples were progressively dehydrated with ethanol (Merck, Germany) and embedded into Epon (EMS, USA) resin. Ultrathin sections (70 nm thick) were stained with uranyl acetate (Merck, Germany) and lead citrate (Merck, Germany), and finally examined with a JEOL 1200EX electron microscope operating at 80 kV.

### Extraction and quantification of long- and short-chain polyphosphates

Aliquots of epimastigote forms (10^7^–10^8^) cultured for 2 days in the absence or presence of 0.3 μM of solenopsins from *S. saevissima* or *S. invicta* were centrifuged to 3,000*g* for 15 min at 4 °C, washed with PBS, and processed for the extraction of either long-chain^69^ or short-chain polyphosphates (polyP)^35^. The long- and short-chain polyP levels were then determined as a function of the amount of orthophosphate (Pi) released upon treatment with an excess of recombinant exopolyphosphatase purified from *Saccharomyces cerevisiae* (scPPX)^32^. The released Pi was measured by the malachite green assay.

### Fluorescent intercalator displacement assay—Evaluation of the affinity of solenopsins with DNA

The DNA fluorescent intercalator displacement assay was based on the protocol as described before^87^. Briefly, samples were prepared in each well of a Costar black 96-well containing 10 mM Tris-HCl pH 7.5, 100 mM NaCl, 4.5 µM ethidium bromide (EtBr, BioRad, USA), and 1.5 µM of EtBr-binding agent salmon sperm DNA (Sigma-Aldrich, USA) in a final volume of 150 µL. Different concentrations of solenopsins (ranging from 0.50 to 15.00 µM as estimated from the average molecular mass calculated from the compositional analysis—see “Results” section), benznidazole (Lafepe, Brazil) (5.0–350.0 µM), miltefosine (Cayman, USA) (5.0–100.0 µM) and of 4′,6-diamino-2-phenylindole (DAPI; Molecular Probes, USA) (1.5–100.0 µM) were added to each experimental condition. Negative control samples had no salmon sperm DNA. After incubation for 30 min at room temperature, each well was read (about five readings) on a Varian Cary Eclipse fluorescence plate reader (λ_Ex_ 545 nm, λ_Em_ 595 nm). Compound assessments were conducted in triplicates (or more) with each well acting as its own control well (no agent = 100% fluorescence; no DNA = 0% fluorescence). Results are presented as the percentage of fluorescence decrease which represents the percentage of intercalator displacement relative to the control wells.

### Evaluation of epimastigotes cell death

In order to evaluate the presence of autophagic vacuoles, parasites (2 × 10^6^ mL^−1^) were cultured in the absence or presence of 0.3 μM of solenopsins for 48 h at 28 °C, centrifuged for 10 min at 1,500*g*, suspended in PBS containing 0.05 mM monodansylcadaverine (MDC, Sigma-Aldrich, USA), and incubated for 1 h at 28°C^33,88^. Positive controls were obtained through similar incubation of parasites in PBS buffer (nutrient deprivation), which induces autophagy in *T. cruzi*^41^. An aliquot of 10% of each cellular suspension was collected, washed in PBS, fixed in 4% formaldehyde-PBS solution (PBS/formaldehyde) for 10 min at room temperature, washed again, and images of the parasites were acquired using an epifluorescence Zeiss AxioPlan II microscope (Oberkochen, Germany). The rest of the cell suspensions (90%) were then washed four times with PBS and suspended in 10 mM Tris-HCl, pH 8 containing 0.1% Triton X-100. Intracellular incorporated MDC was measured by fluorescence photometry (λ_Ex_ 380 nm, λ_Em_ 525 nm) in a Spectra Max 250 micro plate reader (Molecular Devices, USA) and expressed as arbitrary units per number of cells. To normalize the measurements to the number of cells present in each well, a solution of EtBr was added to a final concentration of 0.2 mM and the DNA fluorescence was measured (λ_Ex_ 530 nm, λ_Em_ 590 nm).

Evaluation of programmed cell death induced by solenopsins was performed using the ApopTag® Peroxidase In Situ Apoptosis Detection Kit (EMD Millipore, Germany) following manufacturer’s instructions. In brief, a total of 10^8^ epimastigotes (10^7^ cells mL^−1^ in 10 mL) were cultured in BHI–FCS medium in the absence or presence of solenopsins (2.5 µM of *S. invicta* and 2.5 μM of *S. saevissima*). After 24 h incubation cells were centrifuged at 3,000*g* for 10 min at 4 °C, washed once in PBS buffer and fixed in 1% formaldehyde-PBS solution (PBS/formaldehyde) for 10 min at room temperature. A drop of the cell suspension was dispensed on slides pre-coated with 0.01% poly-l-lysine and, after 10 min, the liquid excess was removed, and the slide washed with PBS buffer. Then, the endogenous peroxidase was inactivated by covering the sections with 3% H_2_O_2_ for 5 min at room temperature. The sections were rinsed with PBS buffer and immersed in terminal deoxynucleotidyl transferase (TdT) buffer (30 mM Trizma base, pH 7.2, 140 mM sodium cacodylate, 1 mM cobalt chloride). TdT (0.3 U µL^−1^) followed by reaction buffer to cover the sections. After incubation in humid atmosphere for 60 min at 37 °C, the reaction was terminated by transferring the slides to TB buffer (300 mM sodium chloride, 30 mM sodium citrate) for 15 min at room temperature. Then the slides were incubated for 30 min with anti-digoxigenin serum (coupled with peroxidase). The slides were washed four times in PBS (for two min each) and the 3,3′-diaminobenzidine (DAB) reagent was added to cover the slides (diluted 50 times in DAB buffer). After incubation for 4–5 min at room temperature, the slides were rinsed with distilled water and counterstained with methyl green (Sigma-Aldrich, USA) for 10 min. The slides were rinsed with water and the samples dehydrated in three washes of butyl alcohol (Sigma-Aldrich, USA) followed by three washes in xylene (Sigma-Aldrich, USA) and processed for light microscopy as above. Results were quantified using CellProfiler image analysis software^89^.

### Statistical analysis

Results presented are from two or three separate experiments, performed in duplicate or triplicate, as indicated. Statistics and plots were generated with R v. 3.0.0^85^, incremented with the open packages “plyr”, “reshape2”, “ggplot2”, “conover.test”, “drc”. Numeric raw data are provided as Supplementary Information 1 with the R scripts (“Supplementary_R_Script_File_Costa_Silva”) to ensure output reproducibility and peer verification of details. Mostly due to limited numbers of repetitions no parameters for data distribution were assumed. Statistical differences using non-parametric Kruskal–Wallis followed by Dunn’s Multiple Comparison Test (in comparing multiple treatments) or by Wilcoxon–Mann–Whitney Test (in comparing two treatments). Our conclusions were also compatible with general patterns obtained by parametric methods (not shown).

## Supplementary information

Supplementary Information.

## Data Availability

The authors state that all materials, data and associated protocols are promptly available to readers without undue qualifications in material transfer agreements.
