# Supplementary material for: Venom alkaloids against Chagas disease parasite: search for effective therapies
Source: Sci Rep. 2020 Jun 30;10:10642. doi: 10.1038/s41598-020-67324-8 (PMC7327076; doi:10.1038/s41598-020-67324-8)
Supplement: Supplementary file 1 — Supplementary Information. [file 41598_2020_67324_MOESM1_ESM.doc]

Venom alkaloids against Chagas disease parasite: Search for effective therapies

Rafael C. M. Costa Silva^1,2^, Eduardo G. P. Fox^1,9^, Fabio M. Gomes^1,3^, Daniel F. Feijó^2^, Isabela Ramos^4,5^, Carolina M. Koeller^1,6^, Tatiana F. R. Costa^1^, Nathalia S. Rodrigues^1^, Ana P. Lima^1^, Georgia C. Atella^4,5^, Kildare Miranda^1,7^, Alejandra C. Schoijet^8^, Guillermo D. Alonso^8*^, Ednildo de Alcântara Machado^1,5*^, & Norton Heise^1*^

**Legends to Supplementary Figures**

**Figure S1. Representative total ion chromatograms (TICs) of the solenopsin alkaloids extracted and purified from the venom of *S. invicta* and *S. saevissima*.** The identification and quantification of the solenopsin alkaloids from the venom of *S. invicta* (INV) **(A)** and *S. saevissima* (SAE) **(B)** was performed by GC-MS as described in M&M. The major peaks highlighted (dashed lines) are Solenopsin A with retention time of 17.95 min and *m/z* 253 (C_11_), Dehydrosolenopsin B with retention time of 19.45 min and *m/z* 279 (C_13_) and Dehydrosolenopsin C with retention time of 20.99 min and *m/z* 307 (C_15_). The first minor peak with retention time of 15.45 min was identified as phthalate (dashed line), a possible minor contaminant of the solvents used for the extraction of the solenopsins.

**Figure S2. Dose-response inhibition curves and the reversible effects of solenopsins in the growth of *Trypanosoma cruzi* epimastigote forms in axenic culture. (A)** Dose-response inhibition curves of Dm-28c epimastigote forms cultured in BHI-FCS medium for 48h with different concentrations of solenopsins from *Solenopsis invicta* (□) and *S.* *saevissima* (○), miltefosine (∆) and benznidazole (◊). **(B)** Dose-response inhibition curves of wild type (void symbols) or overexpressing PI3K (black symbols) CL-Brener epimastigotes cultured as above, with different concentrations of solenopsins from *S.* *invicta* (□) and *S. saevissima* (○). In (A) and (B) the results are expressed as the percentage mean of growth inhibition relative to untreated controls of three separate experiments, each one in duplicates ± s.e.m. **(C)** Epimastigotes (2 × 10^5^.mL^-1^) from the CL-Brener strain were cultured in absence (*) or presence of 0.16 µM (□) or 0.30 µM (○) solenopsins from *S. invicta* and *S. saevissima*, respectively. After 8 days, parasites were collected by centrifugation, washed twice in PBS and re-inoculated in fresh medium at 2 x 10^5^.mL^-1^ for additional 8 days of culture. The results are expressed as the number of cells.mL^-1^ in each day of culture from two independent experiments done in triplicates ± min-max intervals. Statistics: corresponding concentrations at the inflexion point of curves **(A)** and **(B)** were tested with non-parametric Kruskal-Wallis, and treatments are grouped by statistical similarity at alpha = 0.05 where indicated with brackets. For obtained p-values refer to supplementary R script results.

**Figure S3. Fluorescence intercalator displacement histograms at different concentrations of benznidazole, DAPI, miltefosine and solenopsins.** To evaluate the direct interaction of solenopsins with DNA, a solution with fixed concentration of EtBr and salmon sperm DNA were incubated for 30 min at 28ºC in the absence (100% DMSO, -) or presence of increasing concentrations (in μM) of benzonidazole (BENZ, ▼), DAPI (◆), miltefosine (MILT, ▲), and solenopsins from *Solenopsis saevissima* (SAE,

●) and *S. invicta* (INV, ■). Dots are the mean percentage of fluorescence decrease from two independent experiments in triplicates; whiskers are minimum-maximum intervals. Statistics: Comparisons with Kruskal-Wallis at alpha = 0.05 were done among treatments (top) and for internal concentration replicates (bottom). Among treatments DAPI (*) proved statistically different from other treatments. Concentrations which seemed statistically equivalent are marked with a line, and those differing with an *. For details on analyses (e.g. p-values), see the supplementary R script file.

**Figure S4. Cytotoxic effects of solenopsins against mammalian cells.** Cultures of CHO (**A and B**) or BMDM (**C**) cells were prepared in medium (-, +, CTL) or medium supplemented with increasing concentrations of solenopsins (μM) from *Solenopsis invicta* (□, INV) and *S. saevissima* (○, SAE). (**A and C**) Cell culture viability as determined by MTT after 72h of incubation; (**B**) Cell culture viability as determined by LDH after 72h of incubation. Results are mean percentage of cell viability from three and two independent experiments, respectively. Control assays for measurements of complete loss of cell viability (+) were performed after addition of 0.1% TX-100 to the cell cultures. Statistics: on (**A**) treatments with alkaloids did not differ by Wilcoxon’s test and were thus jointly compared with Kruskal-Wallis at alpha = 0.05; the bracket indicates statistically similar treatment results; an asterisk indicates a concentration statistically similar to the positive control (+). For details on analyses (e.g. p-values), see the supplementary R script file.

**Figure S5. Dose-dependent effect of solenopsins in the growth of *Trypanosoma brucei rhodesiense*.** Bloodstream forms of *T. brucei rhodesiense* were cultivated with increasing concentrations of purified solenopsin alkaloids from *S. invicta* or with control diluent for the indicated number of days at 37^o^C and the number of parasites was estimated daily by counting in a Neubauer chamber under the light microscope. The experiments were performed in 3 replicates, at least 2 independent times (*n* = 2) and the results are the mean ± s.e.m. The statistical significance was calculated using Two-way ANOVA and the Bonferroni post-test. The asterisks indicate p<0.0001 from control at day 3.

Fig. S1 (Costa-Silva)


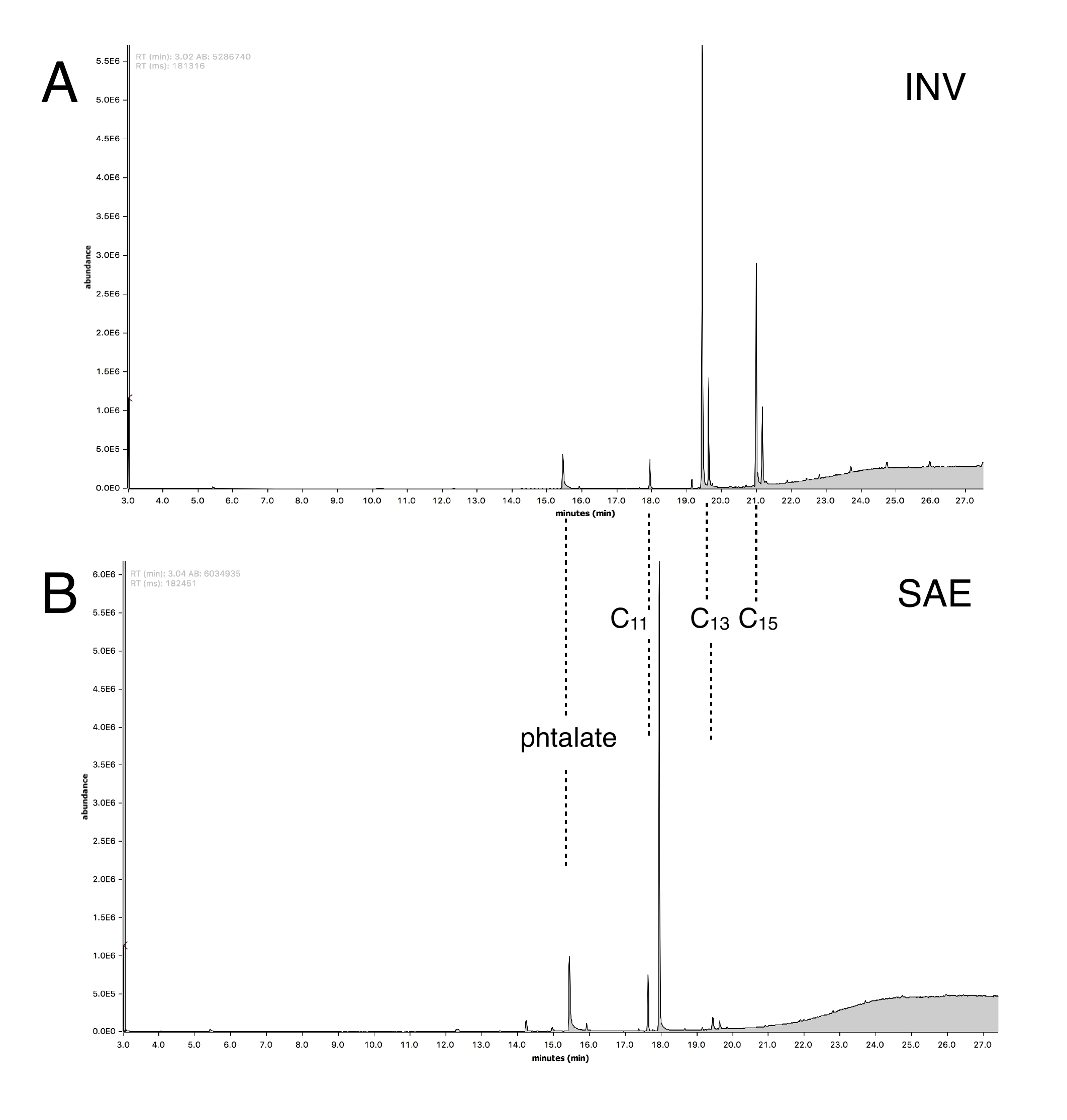


Fig. S2 (Costa_Silva)





Fig. S3 (Costa_Silva)


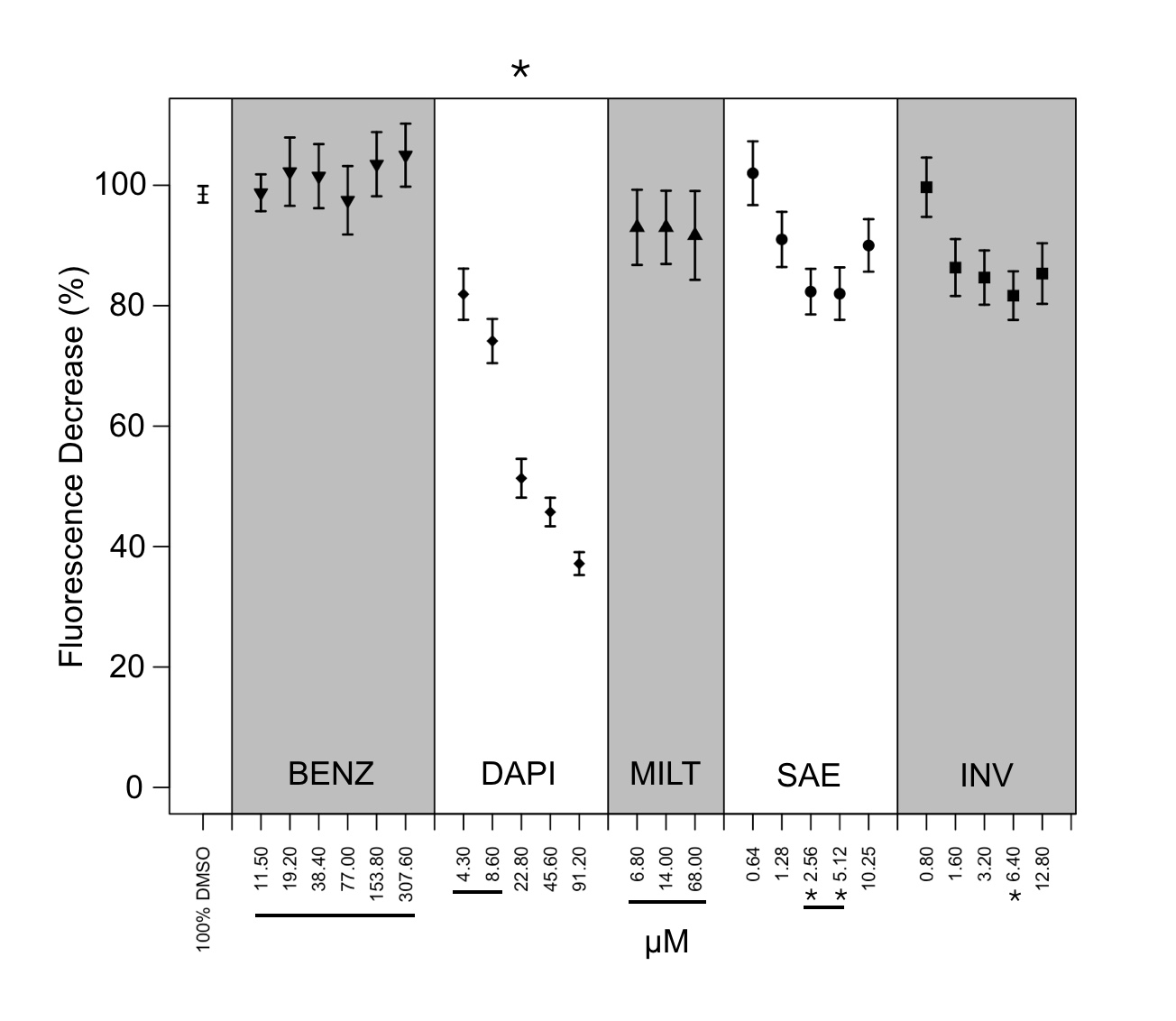


Fig. S4 (Costa_Silva)


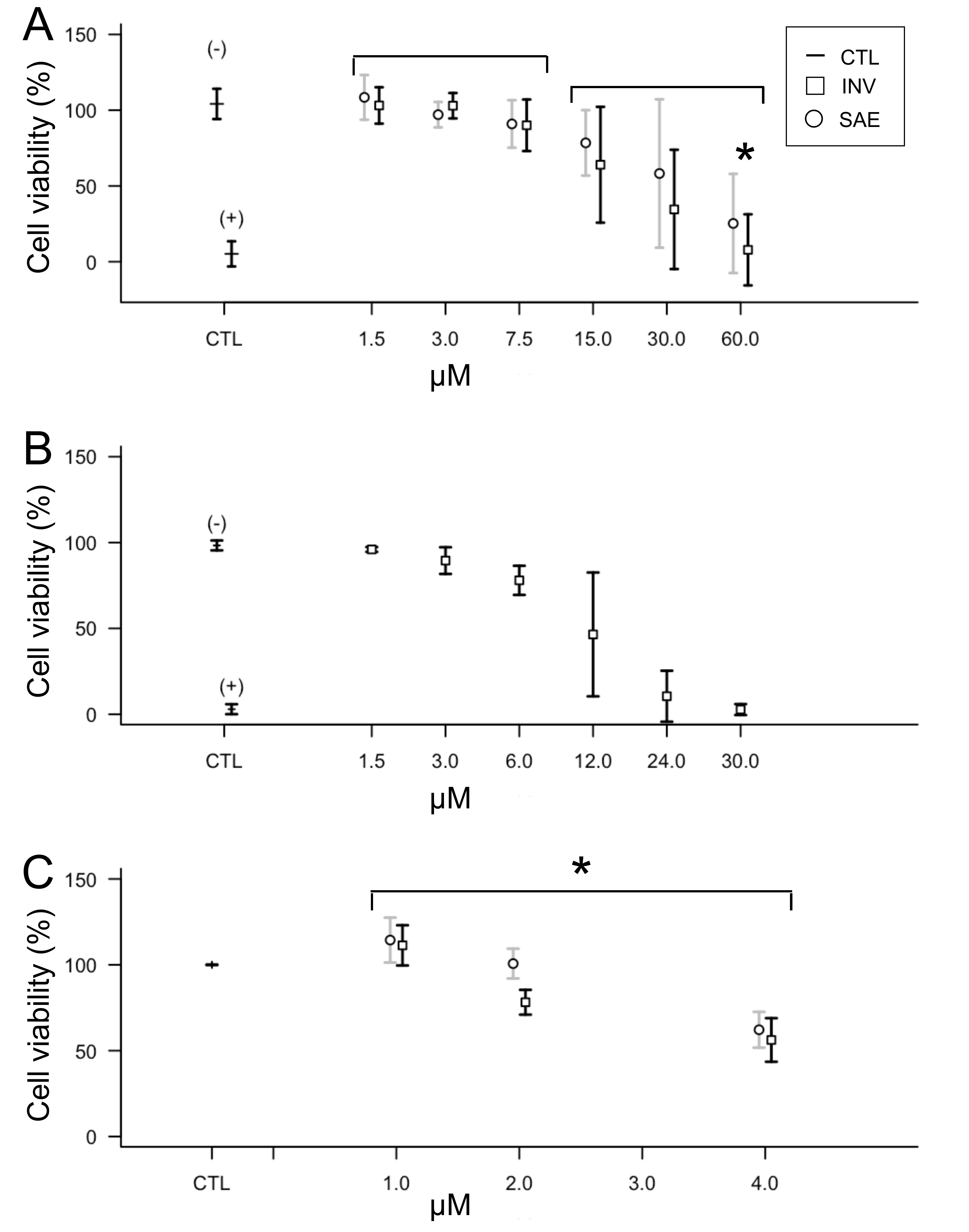


Fig. S5 (Costa_Silva)


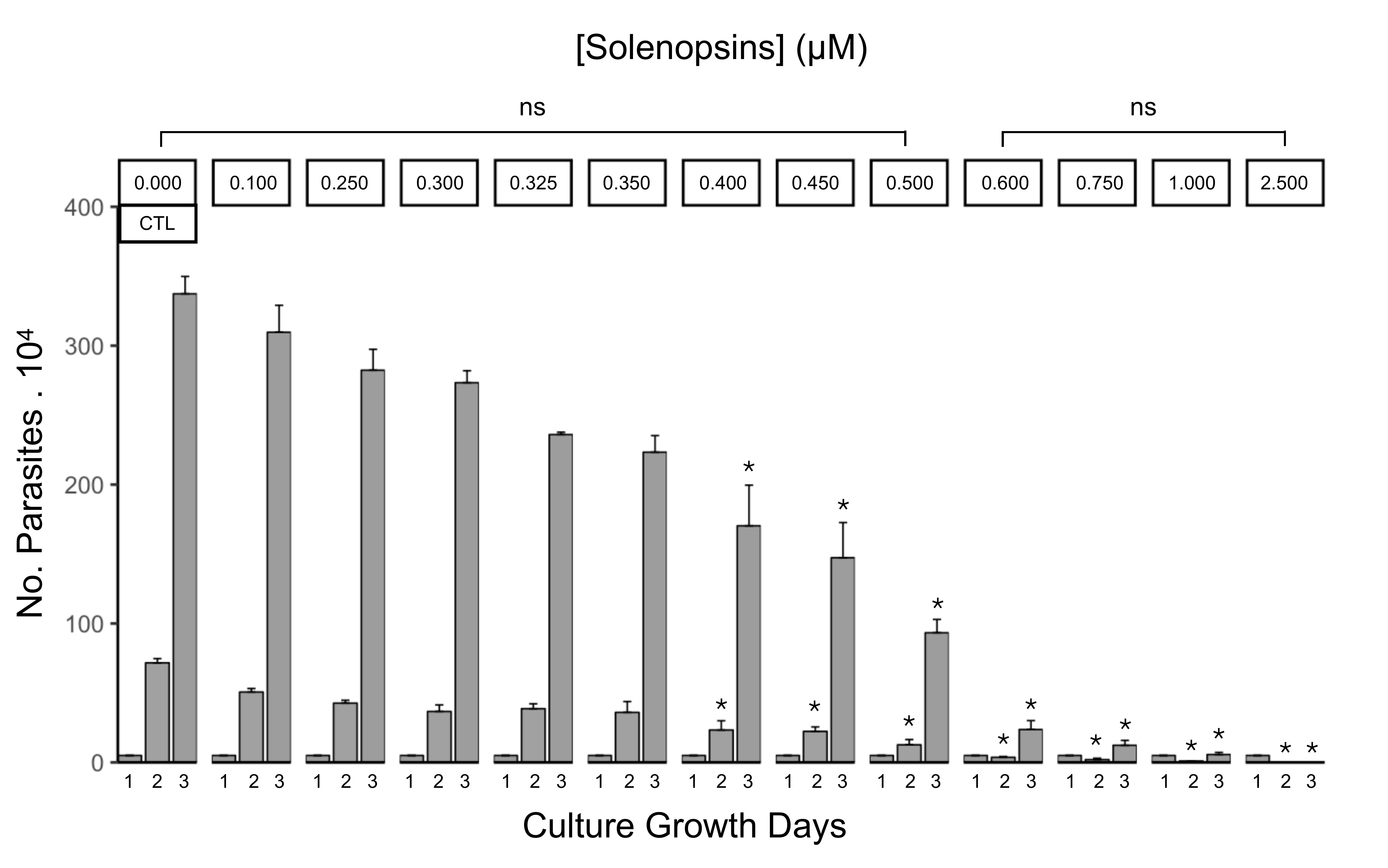


**Scrips to Statistical Analyses**

#********Script written by Eduardo GP Fox with R version 3.5.1 (1st Version: 2018-07-02; Final Version 2020-06-12), RStudio v.1.1.456*********

#Includes Raw Data, Statistical Analyses, and Scripts behind plots for the paper

#Venom alkaloids against Chagas disease parasite: Search for effective therapies, by Costa Silva et al. 2020

#Importing packages for data formatting and preparing plots

require(ggplot2)

require(plyr)

require(reshape2)

require(drc)

require(tidyr)

#Raw data and images posted online as RData objects, to be imported as below:

raw_data<-"https://ndownloader.figshare.com/files/21528132"

load(url(raw_data))

all_figures<-"https://ndownloader.figshare.com/files/23066558"

load(url(all_figures))

# !!! Now Feel free to explore imported plots & raw data from above locally, using R !!!! #

#Statistics compiled from codes given below, per figure and table, along with obtained p-values

#### stats(Figure 1):

#None available; N = 2

#### Stats(Figure 3)

# SUBPART A

#conover.test(PoliP.short.long$value,PoliP.short.long$L1)

# Kruskal-Wallis rank sum test

#

#data: x and group

#Kruskal-Wallis chi-squared = 4.8039, df = 2, p-value = 0.09

#

#

# Comparison of x by group

# (No adjustment)

#Col Mean-|

#Row Mean | INV NEG

#---------+----------------------

# NEG | 1.314118

# | 0.1015

# |

# SAE | 2.253585 1.483429

# | 0.0175* 0.0764

#

#alpha = 0.05

#Reject Ho if p <= alpha/2

#

# SUBPART B

#conover.test(PoliP.long.long$value,PoliP.long.long$L1)

# Kruskal-Wallis rank sum test

#

#data: x and group

#Kruskal-Wallis chi-squared = 20.0983, df = 2, p-value = 0

#

#

# Comparison of x by group

# (No adjustment)

#Col Mean-|

#Row Mean | INV NEG

#---------+----------------------

# NEG | 6.035115

# | 0.0000*

# |

# SAE | -0.802121 -6.679144

# | 0.2147 0.0000*

#

#alpha = 0.05

#Reject Ho if p <= alpha/2

#Note: 9 latest data version

conover.test(melt(Inf_Amas_alk)$value, melt(Inf_Amas_alk)$variable)

#data: x and group

#Kruskal-Wallis chi-squared = 12, df = 4, p-value = 0.02

#

#

####Stats(Figure_5E)

#conover.test(melt(MDC)$value, melt(MDC)$variable)

#No id variables; using all as measure variables

#No id variables; using all as measure variables

# Kruskal-Wallis rank sum test

#

#data: x and group

#Kruskal-Wallis chi-squared = 9.4917, df = 3, p-value = 0.02

#

#

# Comparison of x by group

# (No adjustment)

#Col Mean-|

#Row Mean | BHI INV PBS

#---------+---------------------------------

# INV | -3.676580

# | 0.0031*

# |

# PBS | -7.090547 -3.413967

# | 0.0001* 0.0046*

# |

# SAE | -3.413967 0.262612 3.676580

# | 0.0046* 0.3997 0.0031*

#

#alpha = 0.05

#Reject Ho if p <= alpha/2

####Stats(Figure_6E)

#No id variables; using all as measure variables

#No id variables; using all as measure variables

# Kruskal-Wallis rank sum test

#

#data: x and group

#Kruskal-Wallis chi-squared = 10.7167, df = 3, p-value = 0.01

#

#

# Comparison of x by group

# (No adjustment)

#Col Mean-|

#Row Mean | BHI INV SAE

#---------+---------------------------------

# INV | -4.341103

# | 0.0006*

# |

# SAE | -4.919917 -0.578813

# | 0.0002* 0.2872

# |

# TX_100 | -0.625190 3.393890 3.929767

# | 0.2723 0.0030* 0.0012*

#

#alpha = 0.05

#Reject Ho if p <= alpha/2

####Stats(Figure_7)

# SUBPART A

#data: x and group

#Kruskal-Wallis chi-squared = 12, df = 4, p-value = 0.02

#

#

# Comparison of x by group

# (No adjustment)

#Col Mean-|

#Row Mean | C INV_2.0 INV_4.1 SAE_2.0

#---------+--------------------------------------------

# INV_2.0 | 3.470110

# | 0.0030*

# |

# INV_4.1 | 5.919600 2.449489

# | 0.0001* 0.0171*

# |

# SAE_2.0 | 2.245365 -1.224744 -3.674234

# | 0.0243* 0.1244 0.0021*

# |

# SAE_4.1 | 6.736096 3.265986 0.816496 4.490731

# | 0.0000* 0.0042* 0.2166 0.0006*

#

#alpha = 0.05

#Reject Ho if p <= alpha/2

#

# SUBPART B

#conover.test(melt(BENZ_amastigota)$value, melt(BENZ_amastigota)$variable)

#

#data: x and group

#Kruskal-Wallis chi-squared = 11.5447, df = 4, p-value = 0.02

#

#

# Comparison of x by group

# (No adjustment)

#Col Mean-|

#Row Mean | BENZ_10 BENZ_100 BENZ_2 BENZ_20

#---------+--------------------------------------------

#BENZ_100 | 3.516107

# | 0.0028*

# |

# BENZ_2 | -1.387937 -4.904045

# | 0.0977 0.0003*

# |

# BENZ_20 | 1.480466 -2.035641 2.868403

# | 0.0848 0.0346 0.0084*

# |

# BENZ_C | -2.683345 -6.199453 -1.295408 -4.163811

# | 0.0115* 0.0001* 0.1121 0.0010*

#

#alpha = 0.05

#Reject Ho if p <= alpha/2

#NEWLY PROVIDED DATA

#dunn.test(melt(Inf_Amas_alk)$value, melt(Inf_Amas_alk)$variable)

#dunn.test(melt(Inf_Amas_Benz)$value, melt(Inf_Amas_Benz)$variable)

####stats(Figure_S2)

# SUBPART A

# Comparing replicates

#kruskal.test(L2 ~ value, data=long.Table.6[1:164,], na.action="na.omit")

#Result : Kruskal-Wallis chi-squared = 17.851, df = 19, p-value = 0.5324

#kruskal.test(L2 ~ value, data=long.Table.6[165:328,], na.action="na.omit")

#Result : Kruskal-Wallis chi-squared = 20.457, df = 23, p-value = 0.6142

#kruskal.test(L2 ~ value, data=long.Table.6[329:492,], na.action="na.omit")

#Result : Kruskal-Wallis chi-squared = 21.525, df = 19, p-value = 0.3085

#kruskal.test(L2 ~ value, data=long.Table.6[493:656,], na.action="na.omit")

#Result : Kruskal-Wallis chi-squared = 12.076, df = 14, p-value = 0.6002

# Conclusion: Replicates are equivalent and can be grouped.

#data: x and group

#Kruskal-Wallis chi-squared = 6.9667, df = 3, p-value = 0.07

#

#

# Comparison of x by group

# (No adjustment)

#Col Mean-|

#Row Mean | Invicta PI3K.Inv PI3K.Sae

#---------+---------------------------------

#PI3K.Inv | 2.570568

# | 0.0130*

# |

#PI3K.Sae | 2.807042 0.427158

# | 0.0085* 0.3388

# |

#Saevissi | 2.570568 0.000000 -0.427158

# | 0.0130* 0.5000 0.3388

# SUBPART B

# Comparing replicates

#kruskal.test(L2 ~ value, data=long.Table.10[1:164,], na.action="na.omit")

#Result : Kruskal-Wallis chi-squared = 10.45, df = 12, p-value = 0.5765

#kruskal.test(L2 ~ value, data=long.Table.10[165:328,], na.action="na.omit")

#Result : Kruskal-Wallis chi-squared = 8.2034, df = 12, p-value = 0.769

#kruskal.test(L2 ~ value, data=long.Table.10[329:492,], na.action="na.omit")

#Result : Kruskal-Wallis chi-squared = 41.129, df = 42, p-value = 0.5091

#kruskal.test(L2 ~ value, data=long.Table.10[493:697,], na.action="na.omit")

#Result : Kruskal-Wallis chi-squared = 40.808, df = 35, p-value = 0.2303

# Conclusion: Replicates are equivalent and can be grouped.

#conover.test(melt(LD50.T10)$value, melt(LD50.T10)$variable)

#data: x and group

#Kruskal-Wallis chi-squared = 11.6417, df = 3, p-value = 0.01

#

# Comparison of x by group

# (No adjustment)

#Col Mean-|

#Row Mean | Benznida Invicta Miltefos

#---------+---------------------------------

# Invicta | 5.585280

# | 0.0001*

# |

#Miltefos | 2.902569 -2.898022

# | 0.0072* 0.0072*

# |

#Saevissi | 6.658836 0.579604 3.756266

# | 0.0000* 0.2869 0.0016*

# SUBPART C

# Comparing replicates

#kruskal.test(L2 ~ value, data=long.Table.11[1:54,])

#Result : Kruskal-Wallis chi-squared = 30.917, df = 28, p-value = 0.3208

#kruskal.test(L2 ~ value, data=long.Table.11[55:90,])

#Result : Kruskal-Wallis chi-squared = 29.167, df = 28, p-value = 0.4041

#kruskal.test(L2 ~ value, data=long.Table.10[56:126,])

#Result : Kruskal-Wallis chi-squared = 6.5625, df = 6, p-value = 0.36320

#Conclusion: Replicates are equivalent and can be grouped.

#conover.test(long.Table.6$value,long.Table.6$variable)

#data: x and group

#Kruskal-Wallis chi-squared = 5.9239, df = 3, p-value = 0.12

#

# Comparison of x by group

# (No adjustment)

#Col Mean-|

#Row Mean | Invicta PI3K.Inv PI3K.Sae

#---------+---------------------------------

#PI3K.Inv | 1.110950

# | 0.1341

# |

#PI3K.Sae | 2.427171 1.422529

# | 0.0082* 0.0784

# |

#Saevissi | 1.032925 0.000225 -1.359339

# | 0.1516 0.4999 0.0880

#

#alpha = 0.05

#Reject Ho if p <= alpha/2

#### GENERAL

#Comparing between treatments

#LD50

#conover.test(melt(LD50.T6)$value, melt(LD50.T6)$variable)

####Stats(Figure_S3)

# GENERAL

conover.test(long.Table.10$value,long.Table.10$variable)

# Kruskal-Wallis rank sum test

#data: x and group

#Kruskal-Wallis chi-squared = 31.0629, df = 3, p-value = 0

#

# Comparison of x by group

# (No adjustment)

#Col Mean-|

#Row Mean | Benznida Invicta Miltefos

#---------+---------------------------------

# Invicta | -5.258292

# | 0.0000*

# |

#Miltefos | -1.481719 4.230686

# | 0.0701 0.0000*

# |

#Saevissi | -4.027231 1.340344 -2.903835

# | 0.0000* 0.0909 0.0021*

#

#alpha = 0.05

#Reject Ho if p <= alpha/2

#Specific pairs of datapoints and ranges can be compared by Wilcoxon test

#General usage

#wilcox.test(long.FORMULA.A, long.FORMULA.B)

####Stats(Figure_S4)

# SUBPART A

#conover.test(PoliP.short.long$value,PoliP.short.long$L1)

# Kruskal-Wallis rank sum test

#

#data: x and group

#Kruskal-Wallis chi-squared = 4.8039, df = 2, p-value = 0.09

#

# Comparison of x by group

# (No adjustment)

#Col Mean-|

#Row Mean | INV NEG

#---------+----------------------

# NEG | 1.314118

# | 0.1015

# |

# SAE | 2.253585 1.483429

# | 0.0175* 0.0764

#

#alpha = 0.05

#Reject Ho if p <= alpha/2

#

# SUBPART B

#conover.test(PoliP.long.long$value,PoliP.long.long$L1)

# Kruskal-Wallis rank sum test

#

#data: x and group

#Kruskal-Wallis chi-squared = 20.0983, df = 2, p-value = 0

#

# Comparison of x by group

# (No adjustment)

#Col Mean-|

#Row Mean | INV NEG

#---------+----------------------

# NEG | 6.035115

# | 0.0000*

# |

# SAE | -0.802121 -6.679144

# | 0.2147 0.0000*

#

#alpha = 0.05

#Reject Ho if p <= alpha/2

#### Stats(Table 2)

#Column 1

#dunn.test(melt(Dm28c_values)$value, melt(Dm28c_values)$variable)

# Kruskal-Wallis rank sum test

#

#data: x and group

#Kruskal-Wallis chi-squared = 12.1, df = 3, p-value = 0.01

#

# Comparison of x by group

# (No adjustment)

#Col Mean-|

#Row Mean | Ben INV Mil

#---------+---------------------------------

# INV | 2.781315

# | 0.0027*

# |

# Mil | 1.264911 -1.610235

# | 0.1030 0.0537

# |

# SAE | 3.004163 0.000000 1.739252

# | 0.0013* 0.5000 0.0410

#

#alpha = 0.05

#Reject Ho if p <= alpha/2

#Columns 2 & 3 (tested jointly)

#dunn.test(melt(CL_values)$value, melt(CL_values)$variable)

#Kruskal-Wallis rank sum test

#

#data: x and group

#Kruskal-Wallis chi-squared = 5.633, df = 3, p-value = 0.13

#

# Comparison of x by group

# (No adjustment)

#Col Mean-|

#Row Mean | INV INV_PI3K SAE

#---------+---------------------------------

#INV_PI3K | 2.096882

# | 0.0180*

# |

# SAE | 1.780371 -0.316510

# | 0.0375 0.3758

# |

#SAE_PI3K | 1.807029 -0.134306 0.158725

# | 0.0354 0.4466 0.4369

#

#alpha = 0.05

#Reject Ho if p <= alpha/2

#Column 4

#conover.test(long.Amast$value, long.Amast$variable)

# Kruskal-Wallis rank sum test

#

#data: x and group

#Kruskal-Wallis chi-squared = 5.4222, df = 2, p-value = 0.07

#

# Comparison of x by group

# (No adjustment)

#Col Mean-|

#Row Mean | Ben Inv

#---------+----------------------

# Inv | 3.184011

# | 0.0095*

# |

# Sae | 2.956582 -0.227429

# | 0.0127* 0.4138

#

#alpha = 0.05

#Reject Ho if p <= alpha/2

#Column 6

# Kruskal-Wallis rank sum test

#

#data: x and group

#Kruskal-Wallis chi-squared = 7.4364, df = 2, p-value = 0.02

#

# Comparison of x by group

# (No adjustment)

#Col Mean-|

#Row Mean | Ben INV

#---------+----------------------

# INV | 5.642447

# | 0.0004*

# |

# SAE | 3.507467 -1.997090

# | 0.0049* 0.0430

#

#alpha = 0.05

#Reject Ho if p <= alpha/2

###############################################################################################
